# Serine synthesis through PHGDH coordinates nucleotide levels by maintaining central carbon metabolism

**DOI:** 10.1038/s41467-018-07868-6

**Published:** 2018-12-21

**Authors:** Michael A. Reid, Annamarie E. Allen, Shiyu Liu, Maria V. Liberti, Pei Liu, Xiaojing Liu, Ziwei Dai, Xia Gao, Qian Wang, Ying Liu, Luhua Lai, Jason W. Locasale

**Affiliations:** 1 Department of Pharmacology & Cancer Biology, Duke University School of Medicine, Durham, NC 27710 USA; 2 College of Chemistry and Molecular Engineering, Peking-Tsinghua Center for Life Sciences, Center for Quantitative Biology, Peking University, Beijing, 100871 China

## Abstract

Phosphoglycerate dehydrogenase (PHGDH) catalyzes the committed step in de novo serine biosynthesis. Paradoxically, PHGDH and serine synthesis are required in the presence of abundant environmental serine even when serine uptake exceeds the requirements for nucleotide synthesis. Here, we establish a mechanism for how PHGDH maintains nucleotide metabolism. We show that inhibition of PHGDH induces alterations in nucleotide metabolism independent of serine utilization. These changes are not attributable to defects in serine-derived nucleotide synthesis and redox maintenance, another key aspect of serine metabolism, but result from disruption of mass balance within central carbon metabolism. Mechanistically, this leads to simultaneous alterations in both the pentose phosphate pathway and the tri-carboxylic acid cycle, as we demonstrate based on a quantitative model. These findings define a mechanism whereby disruption of one metabolic pathway induces toxicity by simultaneously affecting the activity of multiple related pathways.

## Abstract (web summary)

Serine synthesis from glucose is required even when serine is available from the environment. Here, the authors explain this paradox by showing that the enzyme PHGDH enables nucleotide synthesis by coordinating anabolic fluxes related to central carbon metabolism, independent of its role in serine production.

## Introduction

The serine, glycine, one-carbon network generates carbon units that satisfy many metabolic demands including nucleotide precursors for anabolic metabolism, redox maintenance, and substrates for methylation reactions that shape the epigenetic landscape^1,2^. Cells derive these one-carbon units from the uptake of serine or from glucose through phosphoglycerate dehydrogenase (PHGDH), the enzyme that catalyzes the committed step towards de novo serine synthesis^3,4^.

Surprisingly, serine uptake from the environment in nearly all contexts far exceeds the demands for one-carbon metabolism. The relative amount of serine synthesized de novo even when cells harbor a PHGDH amplification as observed in cancer is much smaller. Furthermore, supplementation of serine is insufficient to rescue the defects in cellular fitness observed upon PHDGH inhibition^3^. Thus, a major question has been why cells would require PHGDH and the serine synthesis pathway^4^. Proposals have been put forth to account for this conundrum. One is that the transamination reaction downstream of PHGDH that converts glutamate to alpha-ketoglutarate (αKG) is essential. Thus, the resulting αKG that fuels the TCA cycle is the key requirement^3^. Other studies have reported this flux is small relative to other sources that supply αKG^4^. Another proposal is that the small decreases in serine availability from PHGDH inhibition induce a cooperative effect on the folate pool that can impair nucleotide synthesis from exogenous serine^5^. How this may occur is however unknown and may be difficult to reconcile with the current understanding of the kinetics of folate metabolism regulation that indicate a linear response^6^. Fortunately, the advent of selective small-molecule inhibitors targeting PHGDH represent not only potential therapeutics, but also tools to further elucidate how PHGDH maintains cellular fitness^5,7,8^.

In this study, we show that PHGDH is required to maintain nucleotide synthesis by supporting central carbon metabolism. As has been previously reported, PHGDH is found to regulate both de novo purine and pyrimidine biosynthesis. However, this regulation occurs independently of serine synthesis. The underlying mechanism instead is identified to result from disruptions to mass balance. This imbalance compromises the structural support of central carbon metabolism that otherwise allows for the maintenance of both the pentose phosphate pathway and the biosynthetic functions of the tricarboxylic acid (TCA) cycle. Thus, this study identifies the mechanism for why PHGDH is required for nucleotide synthesis independent of serine availability.

## Results

### Inhibition of PHGDH induces broad changes to metabolism

**Fig. 1:**
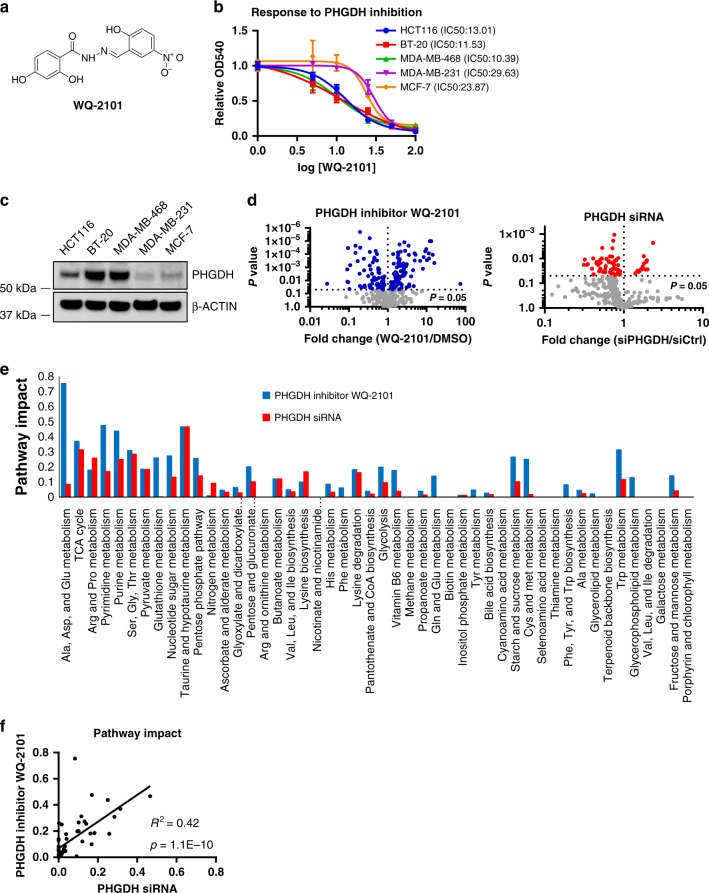
Inhibition of PHGDH induces broad changes in metabolism. **a** Chemical structure of PHGDH inhibitor WQ-2101. **b** Dose-dependent response of cell lines treated with PHGDH inhibitor WQ-2101 for 72 h. Data are the mean of three biological replicates, and error bars represent s.e.m. **c** Immunoblot demonstrating the expression level of PHGDH in cell lines. **d** Volcano plots of metabolites in response to cells treated with 25 μM PHGDH inhibitor WQ-2101 for 24 h (left), or 20 nM siRNA targeting *PHGDH* 72 h post-transfection (right). Colored dots represent metabolites significantly changed between control and treated conditions (*P* < 0.05). Data are mean of three biological replicates. **e** Network-based pathway analysis of statistically significant metabolites altered in each condition. **f** Scatter plot for correlation of metabolic pathway impact scores between WQ-2101 and siRNA targeting *PHGDH*

A previous study identified and validated the selectivity of compounds with PHGDH inhibitory activity and nominated WQ-2101 as a specific, allosteric inhibitor of PHGDH^8^ (Fig. 1a). In agreement, cells cultured with increasing concentrations of WQ-2101 displayed dose-dependent reduction in cell proliferation (Fig. 1b). Consistent with other approaches targeting the serine synthesis pathway^3–5,8^, cells with *PHGDH* amplification or high expression of PHGDH were more sensitive to WQ-2101 due to higher reliance on this pathway (Fig. 1c). We then sought to examine how acute PHGDH inhibition affected global cellular metabolism, which has not to our knowledge been previously reported. To test this, we used Liquid Chromatography coupled to High-Resolution Mass Spectrometry (LC-HRMS) to generate a metabolite profile of HCT116 cells treated either with PHGDH inhibitor WQ-2101 or an siRNA targeting *PHGDH*. In addition to reducing de novo serine synthesis (Supplementary Figure 1a,b), we observed large-scale metabolomic changes in response to PHGDH inhibition (Fig. 1d). A pathway analysis^9^ revealed that the pathways most affected were related to serine-glycine-one carbon (SGOC) metabolism, central carbon metabolism (glycolysis and the TCA cycle), and nucleotide metabolism (Fig. 1e). Although the inhibitory kinetics of a small molecule and siRNA are inherently different and thus we would not expect complete concordance between the genetic and pharmacological approaches, a statistical analysis demonstrated that a majority of the metabolic pathways affected by PHGDH inhibition were consistent among the two approaches, further validating the overall specificity of WQ-2101 and defining the global changes to metabolism upon inhibition of PHGDH (Fig. 1f).

### PHGDH regulates central carbon and nucleotide metabolism

**Fig. 2:**
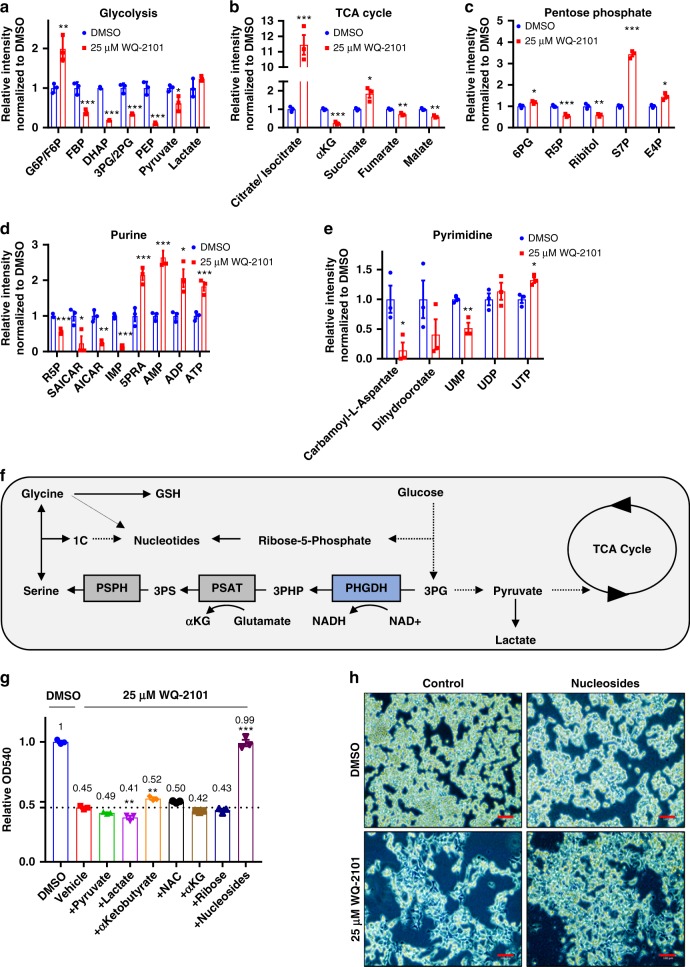
PHGDH inhibition disrupts central carbon and nucleotide metabolism. **a** Relative integrated peak intensities for glycolysis-related metabolites. G6P/F6P (glucose-6-phosphate/fructose-6-phosphate); FBP (fructose-1,6-bisphosphate); DHAP (dihydroxyacetone-phosphate); 3PG/2PG (3-phosphoglycerate/2-phosphoglycerate); PEP (phosphoenolpyruvate). Data are the mean of three biological replicates, and error bars represent s.e.m. *P* < 0.05 [*], *P* < 0.01 [**], *P* < 0.005 [***], Student’s *t*-test. **b** Relative integrated peak intensities for TCA cycle-related metabolites. (αKG) α-ketoglutarate. Data are the mean of three biological replicates, and error bars represent s.e.m. *P* < 0.05 [*], *P* < 0.01 [**], *P* < 0.005 [***], Student’s *t*-test. **c** Relative integrated peak intensities for pentose phosphate pathway-related metabolites. 6PG (6-phosphogluconic acid); R5P (ribose-5-phosphate); S7P (sedoheptulose-7-phosphate); E4P (erythrose-4-phosphate). Data are the mean of three biological replicates, and error bars represent s.e.m. *P* < 0.05 [*], *P* < 0.01 [**], *P* < 0.005 [***], Student’s *t*-test. **d** Relative integrated peak intensities for purine biosynthetic precursors. R5P (ribose-5-phosphate); SAICAR ((S)-2-[5-Amino-1-(5-phospho-d-ribosyl)imidazole-4-carboxamido]succinate); AICAR (5-amino-1-[3,4-dihydroxy-5-(hydroxymethyl)oxolan-2-yl]imidazole-4-carboxamide); IMP (inosine monophosphate); AMP (adenosine monophosphate); ADP (adenosine diphosphate); ATP (adenosine triphosphate). Data are the mean of three biological replicates, and error bars represent s.e.m. *P* < 0.05 [*], *P* < 0.01 [**], *P* < 0.005 [***], Student’s *t*-test. **e** Relative integrated peak intensities for pyrimidine biosynthetic precursors. UMP (uridine monophosphate); UDP (uridine diphosphate); UTP (uridine triphosphate). Cells were treated with 25 μM WQ-2101 for 24 h. Integrated peak intensities were normalized to DMSO control. Data are the mean of three biological replicates, and error bars represent s.e.m. *P* < 0.05 [*], *P* < 0.01 [**], Student’s *t*-test. **f** Schematic of the PHGDH metabolic network. 3PHP (3-phosphohydroxypyruvate); 3PS (3-phosphoserine); 1C (one-carbon unit); GSH (reduced glutathione); PHGDH (phosphoglycerate dehydrogenase); PSAT (phosphoserine aminotransferase); PSPH (phosphoserine phosphatase). **g** MTT assay of cells treated with DMSO control, 25 μM WQ-2101, or 25 μM WQ-2101 supplemented with reagents as indicated for 72 h. OD540 values are relative to DMSO control. Numbers indicate fold change relative to DMSO control. *P* < 0.005 [***], one-way ANOVA. **h** Representative image of cells upon nucleoside rescue. Scale bar = 100 μm

The observation that PHGDH inhibition affected central carbon and nucleotide metabolism promoted us to look more deeply into the mechanism. Indeed, we found HCT116 and BT-20 cells treated with WQ-2101 displayed alterations in glycolysis, TCA cycle, pentose phosphate pathway, and both purine and pyrimidine biosynthesis compared to control (Fig. 2a–e, Supplementary Figure 2, Supplementary Figure 3a–e). Alterations in these metabolic pathways were also observed in cells with *PHGDH* knocked down (Supplementary Figure 4). Numerous metabolic processes are located both upstream and downstream of PHGDH activity (Fig. 2f). These include other pathways connected to and involving glycolysis including NADH/NAD+ redox balance, pentose phosphate pathway, the TCA cycle and the network downstream of serine that involves redox and nucleotide metabolism. To determine which metabolic processes may be involved in the effects of PHGDH inhibition, we supplemented cells with various metabolites related to PHGDH metabolism in the presence of WQ-2101 including pyruvate, lactate, α-ketobutyrate^10^, *N*-acetyl-l-cysteine^11^, cell-permeable α-ketoglutarate, ribose, and nucleosides (Fig. 2g). Only the addition of nucleosides was sufficient to rescue cells from PHGDH inhibition (Fig. 2g, h, Supplementary Figure 3f,g), confirming the major function of PHGDH is to promote nucleotide synthesis as has been reported^5^.

### De novo nucleotide synthesis is altered by PHGDH inhibition

**Fig. 3:**
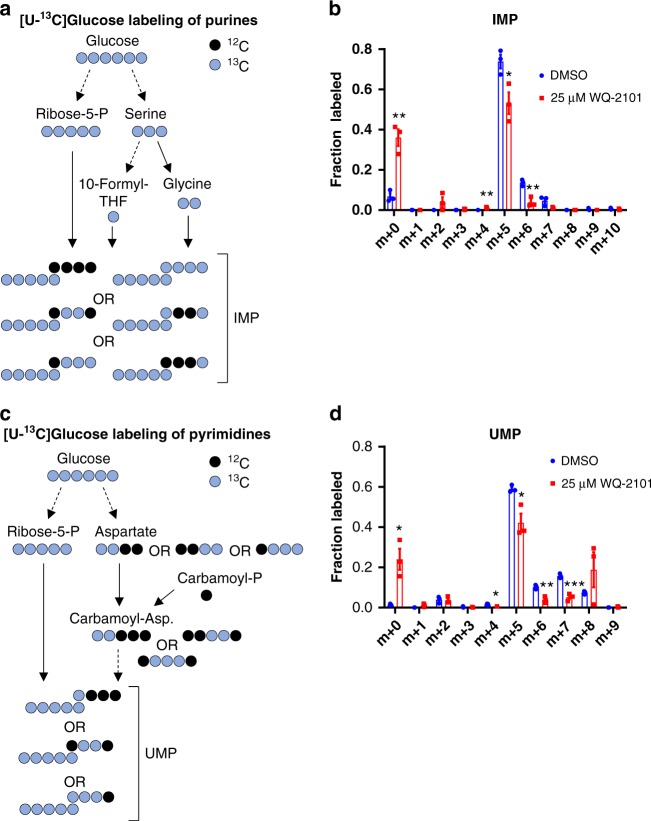
Glucose incorporation into nucleotides is altered upon PHGDH inhibition. **a** Schematic of [U-^13^C] glucose labeling of purines. **b** Mass-isotopomer distribution (MID) of IMP (inosine monophosphate) from [U-^13^C] glucose in cells treated with 25 μM WQ-2101. Data are the mean of three biological replicates, and error bars represent s.e.m. *P* < 0.05 [*], *P* < 0.01 [**], Student’s *t*-test. **c** Schematic of [U-^13^C] glucose labeling of pyrimidines. **d** Mass-isotopomer distribution (MID) of UMP (uridine monophosphate) from [U-^13^C] glucose in cells treated with 25 μM WQ-2101. Data are the mean of three biological replicates, and error bars represent s.e.m. *P* < 0.05 [*], *P* < 0.01 [**], *P* < 0.005 [***], Student’s *t*-test. For labeling experiments, cells were pre-treated with inhibitors for 4 h followed by introduction of [U-^13^C] glucose-containing medium including inhibitors for 20 h

It is thought that the major mechanism of how PHGDH regulates nucleotide synthesis in proliferating cells is by maintaining folate pools and providing glycine for the purine backbone^5^. However, from global metabolite profiling, our results indicate that simultaneous alterations of many other pathways and metabolic processes may contribute to reduced nucleotide synthesis. To study how PHGDH regulates nucleotide metabolism, we used stable isotope tracing by culturing cells in medium supplemented with uniformly labeled glucose ([U-^13^C] glucose), and measured glucose incorporation into numerous pathways that are involved in purine and pyrimidine synthesis with and without PHGDH inhibition via LC-HRMS. Glucose contributes carbons to the purine precursor IMP through ribose-5-phosphate generation in the pentose phosphate pathway, and through glycine and 10-formyl-tetrahydrafolate from the serine synthesis pathway (Fig. 3a). Interestingly, HCT116 cells treated with PHGDH inhibitor WQ-2101 displayed altered isotopomer distributions of IMP (Fig. 3b). For the pyrimidine intermediate UMP, in addition to ribose-5-phosphate, glucose can contribute carbons via aspartate derived from the TCA cycle (Fig. 3c). As with IMP, HCT116 cells treated with WQ-2101 also resulted in altered isotopomer distributions of the pyrimidine intermediate UMP (Fig. 3d). Similar results were obtained in BT-20 cells treated with WQ-2101 and cells treated with a different PHGDH inhibitor compound, NCT-503^5^ (Supplementary Figure 5). Of particular significance, the m+5 isotopomers of IMP and UMP were reduced, demonstrating a reduction in ribose-5-phosphate incorporation from glucose. Importantly, the altered labeling patterns were not the consequence of off-target effects of WQ-2101 as cells with CRISPR-Cas9-mediated knockout of *PHGDH*^8^ treated with WQ-2101 showed labeling patterns consistent with vehicle control treated cells (Supplementary Figure 6). Together, the observed isotope labeling patterns along with our findings that PHGDH ablation affected other glycolysis-related pathways such as the TCA cycle and pentose phosphate pathway show that PHGDH inhibition alters de novo synthesis of both purine and pyrimidine nucleotides through a mechanism that may occur independent of serine-dependent one carbon metabolism.

### Quantitative flux analysis of nucleotide metabolism

**Fig. 4:**
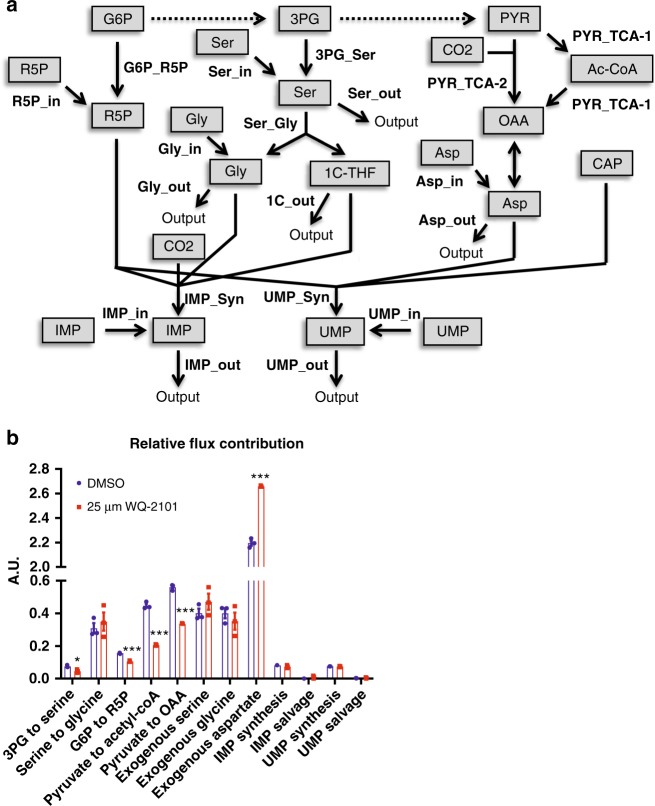
Quantitative flux analysis reveals that PHGDH supports central carbon metabolism. **a** Schematic of metabolic flux analysis model. R5P (ribose-5-phosphate); G6P (glucose-6-phosphate); Ser (serine); Gly (glycine), 3PG (3-phosphoglycerate); CO2 (carbon dioxide); PYR (pyruvate); Ac-CoA (acetyl-coA); OAA (oxaloacetate); Asp (aspartate); CAP (carbamoyl phosphate); UMP (uridine monophosphate); IMP (inosine monophosphate). **b** Relative flux rate of metabolic pathways in cells treated with 25 μM WQ-2101. Data are the mean of three biological replicates, and error bars represent s.e.m. *P* < 0.01 [**], *P* < 0.005 [***], Student’s *t*-test

In general, isotope labeling patterns are not sufficient to infer the extent of metabolic flux through a given pathway^12–16^. Therefore, we developed and applied a quantitative model that computes carbon fluxes into nucleotides with and without PHGDH inhibition from the mass-isotopomer distributions (MIDs) obtained from experiments in Fig. 3. The metabolic network includes flux estimates for 3PG to serine, serine to glycine, glucose-6-phosphate (G6P) to R5P, pyruvate to acetyl-coA, pyruvate to oxaloacetate, exogenous serine input, exogenous glycine input, exogenous aspartate uptake, IMP synthesis, IMP salvage, UMP synthesis, and UMP salvage (Fig. 4a). As expected, our calculations found that PHGDH inhibition reduced carbon flux to nucleotides from the serine synthesis pathway (3PG to serine), although this was a relatively small flux in general (Fig. 4b). Of significance, we found fluxes into nucleotides from the TCA cycle (Pyruvate to Acetyl-CoA and Pyruvate to OAA) and the pentose phosphate pathway (G6P to R5P) were with greater magnitude reduced by PHGDH inhibition relative to the decrease in flux to the serine synthesis pathway (Fig. 4b). Interestingly, the serine to glycine flux into nucleotides was relatively unchanged, suggesting folate availability is uncompromised when the serine synthesis pathway is inhibited (Fig. 4b). This was also observed in BT-20 cells treated with WQ-2101 and cells treated a different PHGDH inhibitor compound, NCT-503^5^ (Supplementary Figure 7). These results demonstrate that PHGDH activity is critical for supporting central carbon metabolism through mass balance. Thus, the results demonstrate that carbon fluxes into nucleotides from the pentose phosphate pathway and TCA cycle were reduced to a greater extent than those in the serine synthesis pathway. In addition, the magnitude of these fluxes were larger than those in the serine synthesis pathway suggesting that disruption of these carbon fluxes may be more detrimental to overall de novo nucleotide synthesis.

### Kinetic profiling of central carbon metabolism

**Fig. 5:**
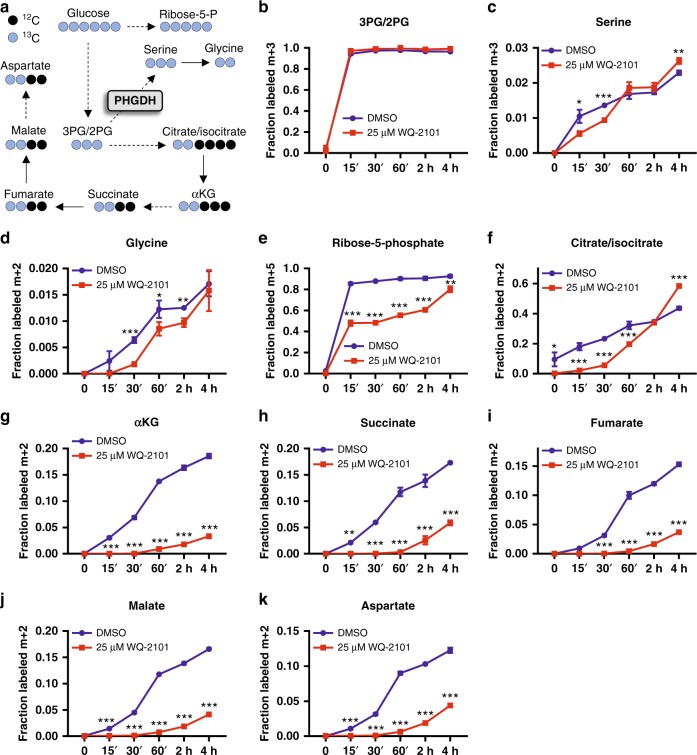
Kinetic profiling confirms reduced flux into the TCA cycle and PPP upon PHGDH inhibition. **a** Schematic of [U-^13^C] glucose labeling of serine, glycine, and central carbon metabolites. **b** Fraction of the m + 3 isotopomer of 3PG/2PG (3-phosphoglycerate/2-phosphoglycerate) labeled in the presence (red) or absence (blue) of 25 μM WQ-2101. 3PG/2PG (3-phosphoglycerate/2-phosphoglycerate). Data are the mean of three biological replicates, and error bars represent s.e.m. **c** Fraction of the m + 3 isotopomer of serine labeled in the presence (red) or absence (blue) of 25 μM WQ-2101. Data are the mean of three biological replicates, and error bars represent s.e.m. *P* < 0.05 [*], *P* < 0.01 [**], *P* < 0.005 [***], Student’s *t*-test. **d** Fraction of the m + 2 isotopomer of glycine labeled in the presence (red) or absence (blue) of 25 μM WQ-2101. Data are the mean of three biological replicates, and error bars represent s.e.m. *P* < 0.05 [*], *P* < 0.01 [**], *P* < 0.005 [***], Student’s *t*-test. **e** Fraction of the m+5 isotopomer of ribose-5-phosphate labeled in the presence (red) or absence (blue) of 25 μM WQ-2101. Data are the mean of three biological replicates, and error bars represent s.e.m. *P* < 0.01 [**], *P* < 0.005 [***], Student’s *t*-test. **f**–**k** Fraction of the m + 2 isotopomers of citrate/isocitrate, αKG (α-ketoglutarate), succinate, fumarate, malate, and aspartate labeled in the presence (red) or absence (blue) of 25 μM WQ-2101. Data are the mean of three biological replicates, and error bars represent s.e.m. *P* < 0.05 [*], *P* < 0.01 [**], *P* < 0.005 [***], Student’s *t*-test. In all experiments, cells were pre-treated with 25 μM WQ-2101 for 24h followed by introduction of [U-^13^C] glucose-containing medium including inhibitor for the indicated times

Given the complexities of flux estimation, we next considered an alternative approach aimed to directly measure flux into metabolic pathways using kinetic flux profiling^17–21^ to further confirm the mechanism. We cultured HCT116 cells in the presence of DMSO or WQ-2101 for 24h in 12C glucose-containing medium, then fed cells [U-^13^C] glucose-containing medium (with the same treatments) and extracted metabolites at numerous time points. We then used LC-HRMS and examined glucose incorporation over time at three different branch points of glycolysis important for nucleotide synthesis: the pentose phosphate pathway (ribose-5-phosphate), serine synthesis pathway (3PG, serine, glycine), and the TCA cycle (citrate/isocitrate, αKG, succinate, fumarate, malate, aspartate) (Fig. 5a). Strikingly, we observed flux through the serine synthesis pathway was largely unchanged in response to PHGDH inhibition (Fig. 5b–d). In contrast, there was a dramatic reduction in flux through the pentose phosphate pathway (Fig. 5e) and the TCA cycle (Fig. 5f–k). Similar reductions in flux through the pentose phosphate pathway and the TCA cycle were found in BT-20 cells treated with WQ-2101 (Supplementary Figure 8) and in cells treated a different PHGDH inhibitor compound, NCT-503^5^ (Supplementary Figure 9). Moreover, [U-^13^C] glutamine tracing of cells treated with DMSO or WQ-2101 revealed labeling patterns consistent with reduced TCA cycle flux upon PHGDH inhibition (Supplementary Figure 10). As with nucleotide labeling patterns, the observed kinetic profiles were not due to off-target effects of WQ-2101 as confirmed with an additional genetic approach (Supplementary Figure 11). Taken together, the metabolic flux analysis and kinetic flux profiling reveal that PHGDH inhibition has a larger effect on central carbon flux than the serine synthesis pathway, and that the mechanism of how PHGDH inhibition leads to reduced nucleotide synthesis is through the loss of mass balance from altered anabolic central carbon flux.

### Restoration of TCA cycle and PPP rescues PHGDH inhibition

**Fig. 6:**
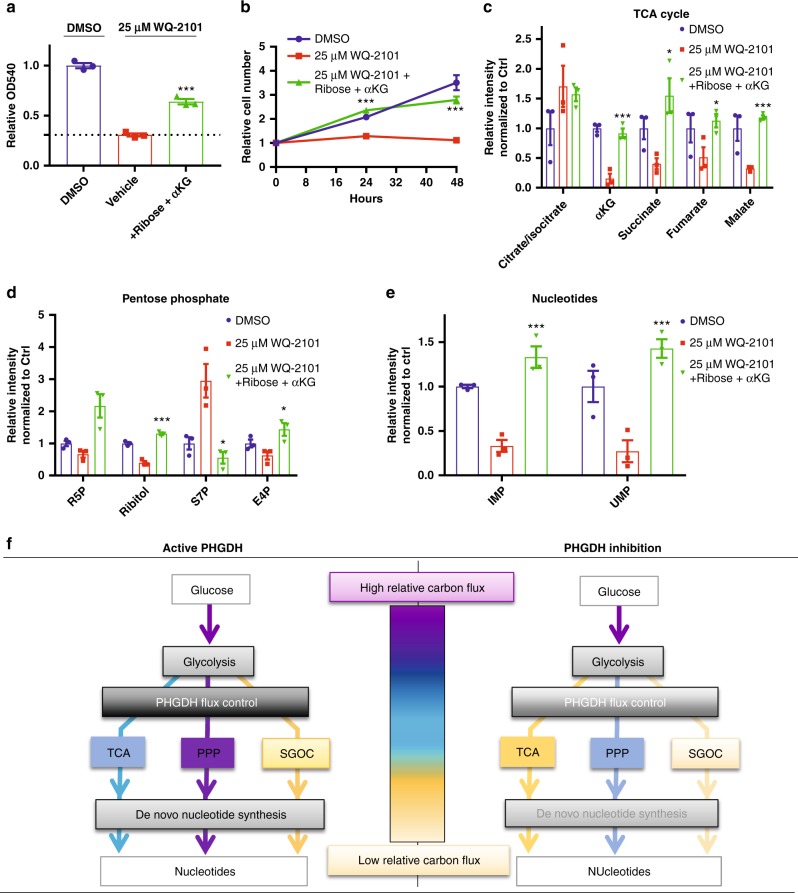
Restoration of TCA and PPP metabolism rescues cell proliferation during PHGDH inhibition. **a** MTT assay of cells treated with DMSO control, 25 μM WQ-2101, or 25 μM WQ-2101 supplemented with ribose and cell-permeable αKG (α-ketoglutarate) for 72 h. OD540 values are relative to DMSO control. Data are the mean of three biological replicates, and error bars represent s.e.m. *P* < 0.005 [***], Student’s *t*-test. **b** Proliferation assay of cells treated with DMSO control, 25 μM WQ-2101, or 25 μM WQ-2101 supplemented with ribose and cell-permeable αKG (α-ketoglutarate) for indicated time points. Cell number is relative to day of treatment (day 0) per condition. Data are the mean of three biological replicates, and error bars represent s.e.m. *P* < 0.005 [***], Student’s *t*-test. **c** Relative integrated peak intensities for TCA cycle-related metabolites. Data are the mean of three biological replicates, and error bars represent s.e.m. *P* < 0.05 [*], *P* < 0.01 [**], *P* < 0.005 [***], Student’s *t*-test. **d** Relative integrated peak intensities for pentose phosphate pathway metabolites. R5P (ribose-5-phosphate); S7P (sedoheptulose-7-phosphate); E4P (erythrose-4-phosphate). Data are the mean of three biological replicates, and error bars represent s.e.m. *P* < 0.05 [*], *P* < 0.005 [***], Student’s *t*-test. **e** Relative integrated peak intensities for IMP (inosine monophosphate) and UMP (uridine monophosphate). Data are the mean of three biological replicates, and error bars represent s.e.m. *P* < 0.01 [**], Student’s *t*-test. **f** Schematic for how PHGDH regulates glucose carbon flux control (left) and effects on these flux controls upon its inhibition (right). TCA (TCA cycle); PPP (pentose phosphate pathway); SGOC (serine, glycine, one-carbon metabolism). Purple to yellow color scale indicates high to low relative carbon flux through a given metabolic pathway

We then asked whether simultaneous restoration of pentose phosphate pathway and TCA cycle metabolism could rescue cells from PHGDH inhibition. Indeed, supplementing cells treated with WQ-2101 with ribose and cell-permeable α-ketoglutarate was sufficient to attenuate the proliferative defects of PHGDH inhibition (Fig. 6a, b, Supplementary Figure 12a, b), while supplementation with ribose and the one-carbon donor formate was not, consistent with the hypothesis that one-carbon unit availability is uncompromised during PHGDH inhibition (Supplementary Figure 12c). Furthermore, we performed LC-HRMS on cells under the rescue conditions and observed an almost complete reversal in TCA cycle (Fig. 6c) and pentose phosphate pathway (Fig. 6d) metabolites. Importantly, we also found cells with PHGDH inhibition supplemented with ribose and cell-permeable α-ketoglutarate had restored levels of purine (IMP) and pyrimidine (UMP) precursors (Fig. 6e). In all, our data supports the mechanism whereby PHGDH regulates the mass balance of central carbon metabolism and thus controls the fluxes into the TCA cycle and pentose phosphate pathway to a greater extent than the SGOC network. Thus, the nucleotide deficiencies in response to PHGDH inhibition are largely due to reductions in central carbon flux (Fig. 6f).

## Discussion

The current paradigm is that the major function of PHGDH in proliferating cells is to maintain folate pools for nucleotide synthesis and contribute to the glycine backbone of purines^5^. In our study, we confirm that PHGDH is critical for nucleotide synthesis, and define a new mechanism by which PHGDH contributes to and is required for nucleotide metabolism. Namely, PHGDH regulates and is required for nucleotide metabolism by supporting the backbone of central carbon metabolism. Thus, diversion of glycolysis into serine synthesis also coordinates anabolic fluxes related to central carbon metabolism^22^. Notably, these fluxes controlled by PHGDH include ribose synthesis from the pentose phosphate pathway and the nucleobases from the TCA cycle. Supporting this model, our calculations using metabolic flux analysis and measurements using kinetic flux profiling demonstrate that the major fluxes affected upon PHGDH inhibition that alter nucleotide metabolism are related to central carbon metabolism, and not the serine synthesis pathway (Figs. 4 and 5). Our findings are thought to be a direct mechanism of PHGDH inhibition rather than a general consequence of reduced cell proliferation, consistent with previous reports showing metabolic flux and metabolite availability may not coordinate with proliferation status^22,23^.

Furthermore, these findings define a mechanism whereby the diversion of flux into one pathway is coupled to and thus controls numerous other pathways through long range interactions. This regulation has been in shown in other cases to occur via allosteric mechanisms^24^ where changes in the concentration of metabolites in one pathway bind to enzymes and regulate the activity of other pathways. This study provides an additional example of how such a long range regulatory mechanism in metabolism can be achieved. In this study, however, it is the mass balance and the requirement of maintaining flux balance is sufficient to regulate the flux into other neighboring anabolic pathways.

Conversely, other mechanisms have been proposed that lead to increased serine synthesis by accumulation of glycolytic intermediates^25–27^. In these scenarios, serine synthesis would serve additional biological functions by also coupling flux into related anabolic pathways. Furthermore, disruption of any of these mechanisms that push flux into serine synthesis would also affect both mitochondrial and pentose phosphate metabolism. Thus, these findings underscore the need for quantitative flux analysis to understand the metabolic requirements of altered enzyme activity, which may not be apparent from measurements of metabolite levels, and ultimately in understanding unanticipated contexts in which targeting PHGDH might be effective in proliferative disease.

## Methods

### Cell culture and reagents

HCT116, MDA-MB-468, and MCF-7 cells were purchased from ATCC. BT-20 and MDA-MB-231 cells were kindly provided by Dr. Donald McDonnell (Duke University). SCOV3 CRISPR-Cas9 mediated *PHGDH* knockout cells were generated as previously described^8^. All cells were maintained in RPMI 1640 (GIBCO) supplemented with 10% heat-inactivated fetal bovine serum (Sigma, F2442) and 100 U/mL penicillin 100 mg/mL streptomycin (GIBCO). Cells were cultured in a 37 °C, 5% CO_2_ atmosphere. Cell lines were authenticated by their source using short-tandem repeat (STR) profiling, and tested negative for mycoplasma contamination. MCF-7 and BT-20 are listed in the ICLAC database of commonly misidentified cells; the use of these cell lines is justified by STR profiling authentication and their respective PHGDH levels (Fig. 1c). For isotope tracing experiments, dialyzed fetal bovine serum (Thermo Fisher Scientific, 88440) was substituted for fetal bovine serum upon administration of the isotope-containing medium. WQ-2101 was synthesized as previously described^8^. Sodium DL-lactate (71720), Sodium α-Ketobutyrate (K0875), Dimethyl-α-ketoglutarate (cell-permeable α-ketoglutarate) (349631), *N*-Acetyl-l-cysteine (A9165), D-Ribose (R9629), and NCT-503 (SML1659) were purchased from Sigma. Formic acid was purchased from Fischer Scientific (A117). Sodium pyruvate was purchased from Santa Cruz Biotechnology (sc-208397A). EmbryoMax Nucleosides was purchased from Millipore (ES-008-D). U-^13^C-glucose and U-^13^C-glutamine were purchased from Cambridge Isotope Laboratories (CLM-1396-10 and CLM-1822-H-PK).

### MTT and proliferation assays

MTT (3-[4,5-Dimethylthiazol-2-yl]-2,5-diphenyltetrazolium bromide) was purchased from Thermo Fisher Scientific (M6494). Cells were seeded at a density of 2-5 × 10^3^ cells per well in 96-well plates and allowed to adhere for 24 h prior to treatment. Following treatment, medium was carefully aspirated and replaced with 100 μL of 0.5 mg/mL MTT solution in phenol red-free RPMI 1640 (GIBCO) and incubated at 37 °C for 1.5 h. Following incubation, MTT solution was carefully removed and 200 μL DMSO was added to dissolve the formazan. Absorbance was recorded at 540 nm. For proliferation assays, cells were seeded at a density of 2.5 × 10^4^ cells per well in 12-well plates and allowed to adhere for 24 h prior to treatment. Cells were then counted for each condition (day 0) prior to administration of indicated treatments, followed by cell counting at indicated time points using a MOXI Z automated cell counter (Orflo).

### Immunoblotting

Cells were lysed in RIPA buffer (VWR International) containing freshly added protease inhibitor complex (Roche). 20 μg of protein was loaded on precast NuPAGE Bis-Tris gels (Thermo Fisher Scientific) followed by transfer onto nitrocellulose. Chemiluminescent signals were detected with Western Lighting Plus-ECL (Perkin Elmer, NEL103001) and imaged with the ChemiDoc Touch Imaging System (Bio-Rad). Anti-PHGDH antibody was purchased from Sigma (WH0026227M1), and was used at 1:1000 dilution. Anti-β-ACTIN antibody was purchased from Cell Signaling (3700S), and was used at 1:2000 dilution. Uncropped western blot images are reported in Supplementary Figure 13.

### Microscopy

Cells were seeded at a density of 2–5 × 10^3^ cells per well in 96-well plates and allowed to adhere for 24 h prior to treatment. Following treatment, medium was replaced with 1× PBS prior to imaging. Images were captured using a Leica DM IL LED microscope equipped with a Leica MC170HD camera at ×10 objective using LAS EZ software (Leica). Scale bars = 100 μm.

### siRNA transfections

ON-TARGETplus Non-targeting control siRNA (D-001810-01-05) and SMARTpool ON-TARGETplus siRNA oligonucleotides targeting human *PHGDH* (L-009518-00) were purchased from Dharmacon. siRNA transfections were performed using Lipofectamine RNAiMAX (Thermo Fisher Scientific) according to the manufacturer’s protocol. Experiments were performed 72 h post transfection.

### Metabolite extraction

Polar metabolite extraction was conducted as previously described^28,29^. Briefly, 1.5–3 × 10^5^ cells per well were seeded into six-well plates and allowed to adhere for 24 h prior to treatment. Cell confluence was equal across conditions at the time of extraction. Following treatment, medium was aspirated and 1 mL of extraction solvent (80% methanol/water) cooled to −80 °C was immediately added to each well prior to transferring the plates to −80 °C for 15 min. The plates were then removed, placed on dry ice, and the cells were scraped into the extraction solvent and transferred to Eppendorf tubes. Metabolite extractions were centrifuged at 20,000 × *g* at 4 °C for 10 min. The solvent in each sample was then transferred to a new Eppendorf tube and evaporated using a speed vacuum. For polar metabolite analysis, the evaporated cell extracts were first dissolved in 15 μL water and then diluted with 15 μL methanol/acetonitrile (1:1 v/v). Finally, samples were centrifuged at 20,000 × *g* at 4 °C for 10 min, and the supernatants were transferred to LC vials prior to HPLC injection (3 μL).

### High-performance liquid chromatography

Compound separation was performed using an XBridge amide column (100 × 2.1 mm i.d., 3.5 μm; Waters) on a Dionex Ultimate 3000 UHPLC at room temperature. Mobile phase A: water with 5 mM ammonium acetate, pH 6.9; Mobile phase B: 100% acetonitrile. The following linear gradient was used: 0 min, 85% B; 1.5 min, 85% B; 5.5 min, 35% B; 10 min, 35% B; 10.5 min, 10% B; 12.5, 10% B; 13.5 min, 85% B; 20 min, 85% B. Flow rate was 0.15 ml/min from 0 to 5.5 min, 0.17 ml/min from 6.9 to 10.5 min, 0.3 ml/min from 10.6 to 17.9 min, and 0.15 ml/min from 18 to 20 min. All solvents used were LC-MS grade, and were purchased from Fisher Scientific.

### Mass spectrometry

Mass spectrometry was performed using the Q Exactive plus (Thermo Scientific) instrument as previously described^28,29^. The Q Exactive Plus MS is equipped with a heated electrospray ionization probe (HESI) and the relevant parameters are as follows: evaporation temperature, 120 °C; sheath gas, 30; auxiliary gas, 10; sweep gas, 3; spray voltage, 3.6 kV for positive mode and 2.5 kV for negative mode. Capillary temperature was set at 320 °C, and S lens was 55. A scan range from 70 to 900 (*m*/*z*) was used. Resolution was set at 70,000. The maximum injection time was 200 ms, and the automated gain control was targeted at 3 × 10^6^ ions.

### Metabolite peak extraction and data analysis

Raw peak data was processed on Sieve 2.0 software (Thermo Scientific) with peak alignment and detection performed according to the manufacturer’s protocol. The method “peak alignment and frame extraction” was applied for targeted metabolite analysis. An input file of theoretical *m*/*z* and detected retention time was used for targeted metabolite analysis, and the *m*/*z* width was set to 5 ppm. An output file was obtained after data processing that included detected *m*/*z* and relative intensity in the different samples. For isotope tracing experiments, the mass isotopomer distributions were calculated and normalized by comparing the ratio of labeled to unlabeled metabolites in each sample. For ribose and αKG rescue experiments, peak intensities for ribose + αKG + inhibitor were normalized to ribose + αKG + vehicle. Metabolite pathway impacts were determined by MetaboAnalyst pathway analysis [www.metaboanalyst.ca] using the following parameters: Over Representation Analysis- Hypergeometric Test; Pathway Topology Analysis- Relative-betweeness Centrality. Volcano plots were generated using GraphPad Prism 7.

### Metabolic flux analysis

A metabolic model including 19 reactions and 21 metabolites in pentose phosphate pathway, serine synthesis, TCA cycle and nucleotide synthesis pathway was used. Biomass synthesis was modeled based on literature values for molecular composition of dry cell weight^30^. The model was then converted to elementary metabolite units (EMUs)^31^ according to the stoichiometry and carbon atom mapping relationships. The EMU model was used in estimation of the fluxes from mass isotopomer distributions (MIDs) of metabolites measured by LC-HRMS. Flux ratios were solved from MIDs of metabolites at the branch points. Metabolic fluxes relative to growth rate were then solved analytically from the combination of flux ratios at branch points and the stoichiometric matrix. Mean and standard deviation values were computed based on fluxes estimated from three biological replicates. Construction of the metabolic flux analysis model is reported in Supplementary Note 1.

### Statistics

All experiments contained three biological replicates. Results shown as means; error bars represent the standard error of the mean. The unpaired Student’s *t*-test was used to determine statistical significance of differences between means (*P* < 0.05 [*], *P* < 0.01 [**], *P* < 0.005 [***]) unless otherwise stated.

### Code availability

Source code for the metabolic flux analysis model is available on GITHUB via https://github.com/LocasaleLab/Reid-et-al-2018.

### Reporting summary

Further information on experimental design is available in the Nature Research Reporting Summary linked to this article.

## Supplementary information

Supplementary Information

Peer Review File

Reporting Summary

## Data Availability

All metabolomics and metabolic flux analysis data sets are available on GITHUB via https://github.com/LocasaleLab/Reid-et-al-2018. A reporting summary for this Article is available as a Supplementary Information file. All other data supporting the findings of this study are available from the corresponding author on reasonable request.
