# Supplementary material for: Serine synthesis through PHGDH coordinates nucleotide levels by maintaining central carbon metabolism
Source: Nat Commun. 2018 Dec 21;9:5442. doi: 10.1038/s41467-018-07868-6 (PMC6303315; doi:10.1038/s41467-018-07868-6)
Supplement: Supplementary file 1 — Supplementary Information [file 41467_2018_7868_MOESM1_ESM.pdf]

Serine synthesis through PHGDH coordinates nucleotide levels by maintaining central carbon metabolism

Reid et al.

Supplementary Information

Supplementary Figures 1-13
Supplementary Note 1

Supplementary Figure 1: Serine synthesis under WQ-2101 treatment and *PHGDH* siRNA

a

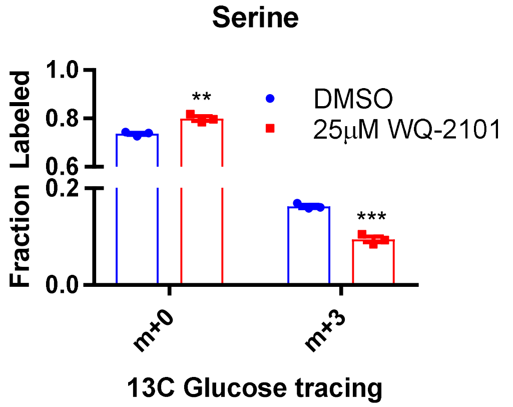

b

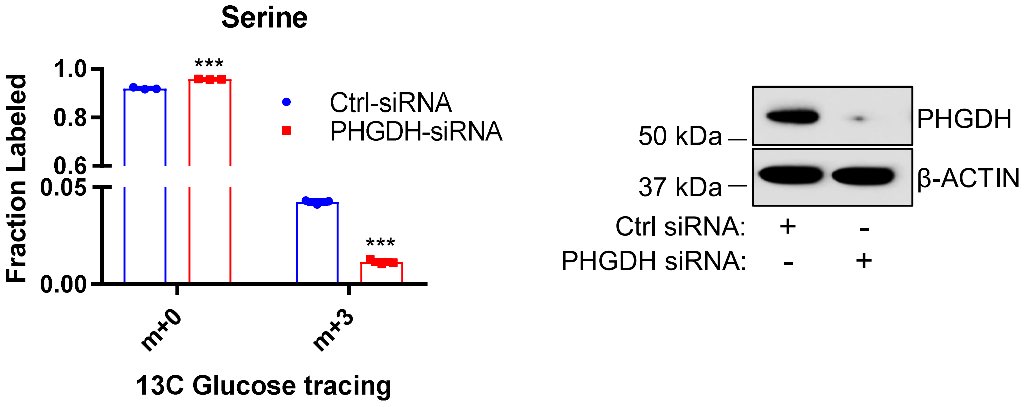

Supplementary Figure 1: Serine synthesis under WQ-2101 treatment and *PHGDH* siRNA. (a) Cells were pre-treated with DMSO control or WQ-2101 for 4h followed by ^{13}C-glucose tracing for 20h in the presence of DMSO or WQ-2101. Data are the mean of three biological replicates, and error bars represent s.e.m. $P < 0.01$ [**], $P < 0.005$ [***], Student's *t*-test. (b) Cells were transfected with 20 nM control siRNA or 20nM siRNA targeting *PHGDH* and 72h post-transfection ^{13}C-glucose tracing was performed for 6h. Data are the mean of three biological replicates, and error bars represent s.e.m. $P < 0.005$ [***], Student's *t*-test.

Supplementary Figure 2: Central carbon and nucleotide metabolism at early time point

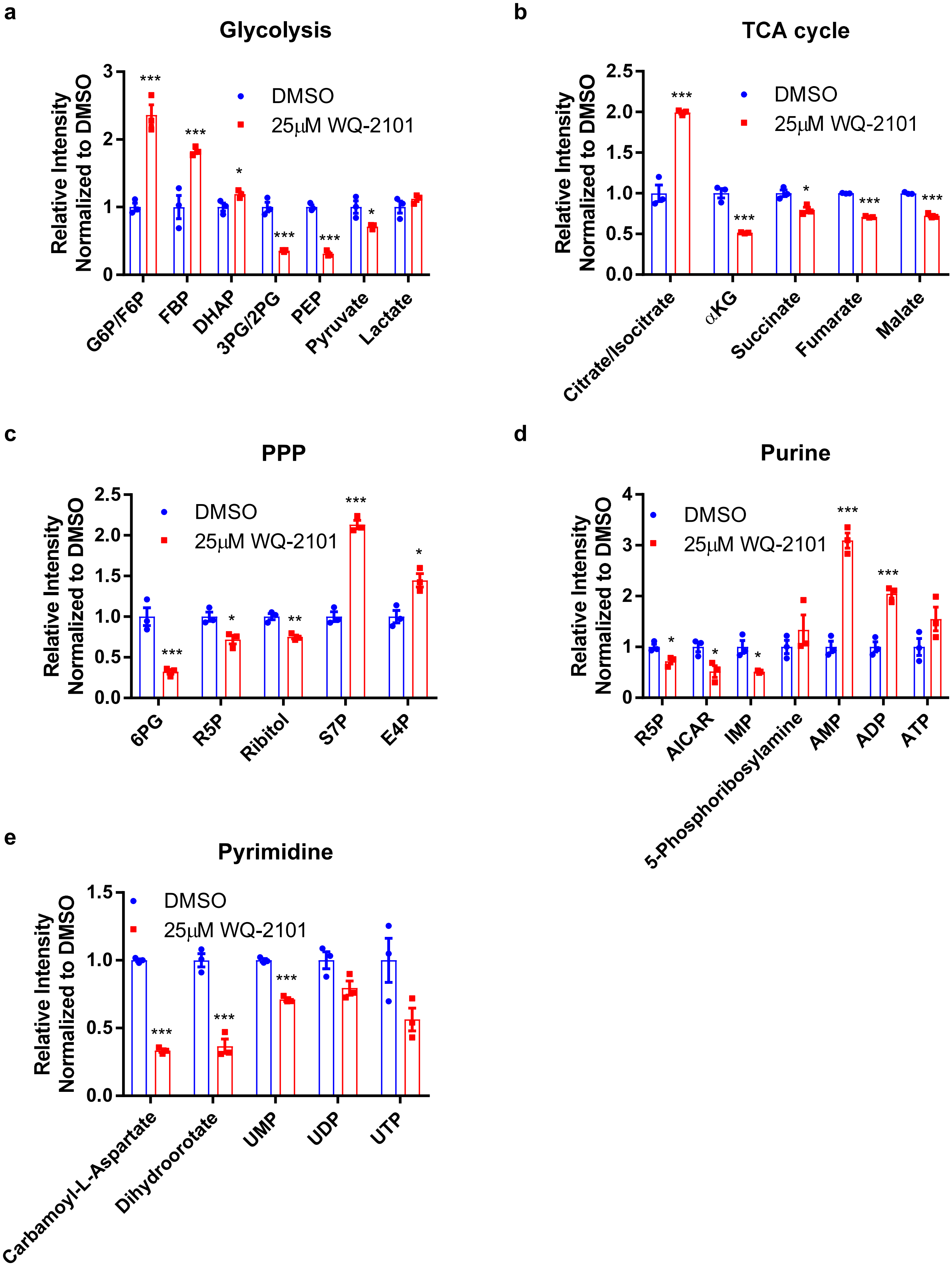

Supplementary Figure 2: Central carbon and nucleotide metabolism at early time points. (a) Relative integrated peak intensities for glycolysis-related metabolites. G6P/F6P (glucose-6-phosphate/fructose-6-phosphate); FBP (fructose-1,6-bisphosphate); DHAP (dihydroxyacetone-phosphate); 3PG/2PG (3-phosphoglycerate/2-phosphoglycerate); PEP (phosphoenolpyruvate). Data are the mean of three biological replicates, and error bars represent s.e.m. $P < 0.05$ [*], $P < 0.005$ [***], Student's *t*-test. (b) Relative integrated peak intensities for TCA cycle-related metabolites. (αKG) α-ketoglutarate. Data are the mean of three biological replicates, and error bars represent s.e.m. $P < 0.05$ [*], $P < 0.005$ [***], Student's *t*-test. (c) Relative integrated peak intensities for pentose phosphate pathway-related metabolites. 6PG (6-phosphogluconic acid); R5P (ribose-5-phosphate); S7P (sedoheptulose-7-phosphate); E4P (erythrose-4-phosphate). Data are the mean of three biological replicates, and error bars represent s.e.m. $P < 0.05$ [*], $P < 0.01$ [**], $P < 0.005$ [***], Student's *t*-test. (d) Relative integrated peak intensities for purine biosynthetic precursors. R5P (ribose-5-phosphate); AICAR (5-amino-1-[3,4-dihydroxy-5-(hydroxymethyl)oxolan-2-yl]imidazole-4-carboxamide); IMP (inosine monophosphate); AMP (adenosine monophosphate); ADP (adenosine diphosphate); ATP (adenosine triphosphate). Data are the mean of three biological replicates, and error bars represent s.e.m. $P < 0.05$ [*], $P < 0.005$ [***], Student's *t*-test. (e) Relative integrated peak intensities for pyrimidine biosynthetic precursors. UMP (uridine monophosphate); UDP (uridine diphosphate); UTP (uridine triphosphate). Data are the mean of three biological replicates, and error bars represent s.e.m. $P < 0.005$ [***], Student's *t*-test. For all panels, cells were treated with DMSO or 25μM WQ-2101 for 6h. Integrated peak intensities were normalized to DMSO control.

Supplementary Figure 3: BT-20 central carbon and nucleotide metabolism

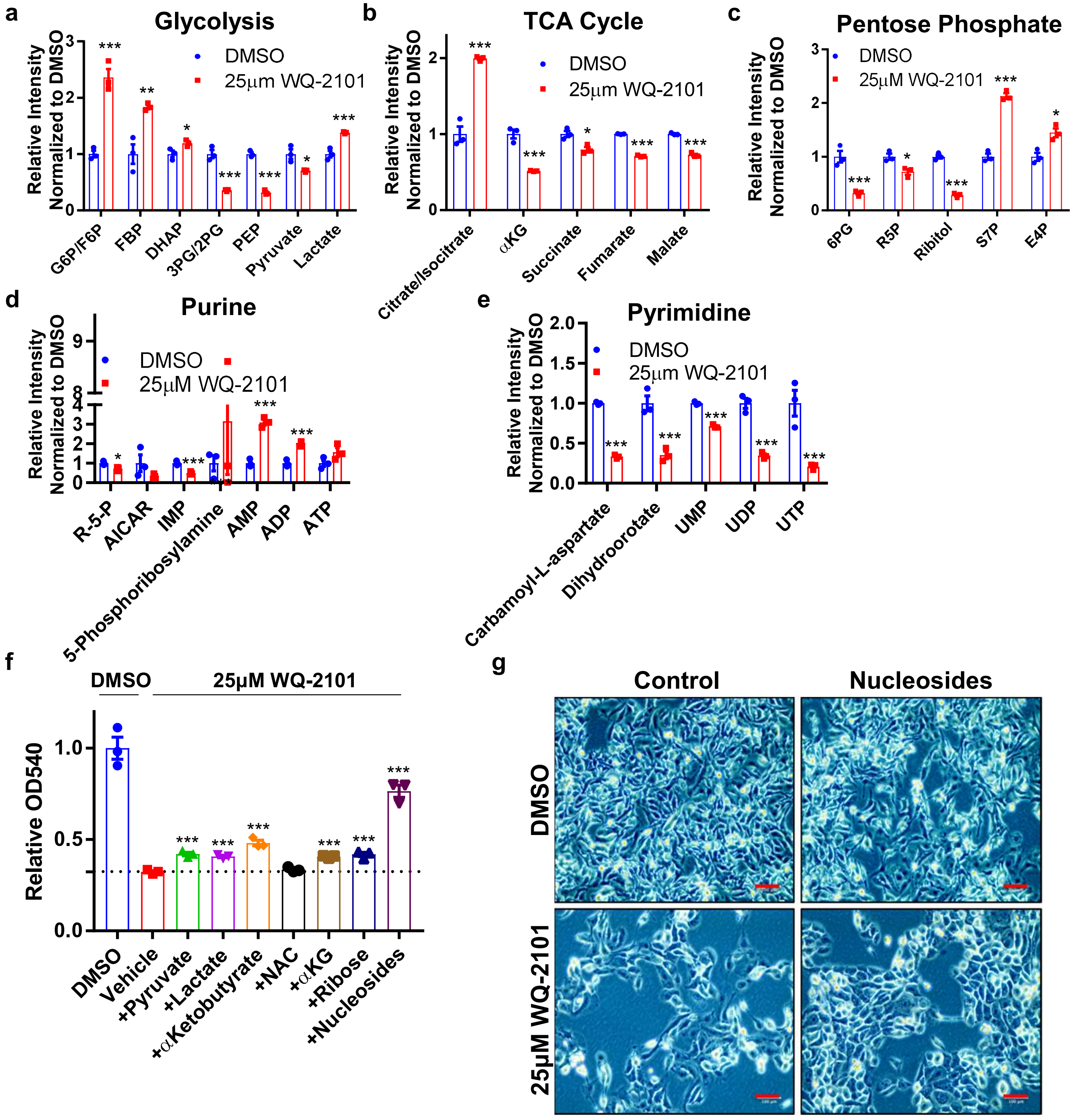

Supplementary Figure 3: BT-20 central carbon and nucleotide metabolism.

(a) Relative integrated peak intensities for glycolysis-related metabolites. G6P/F6P (glucose-6-phosphate/fructose-6-phosphate); FBP (fructose-1,6-bisphosphate); DHAP (dihydroxyacetone-phosphate); 3PG/2PG (3-phosphoglycerate/2-phosphoglycerate); PEP (phosphoenolpyruvate). Data are the mean of three biological replicates, and error bars represent s.e.m. $P < 0.05$ [*], $P < 0.01$ [**], $P < 0.005$ [***], Student's *t*-test. (b) Relative integrated peak intensities for TCA cycle-related metabolites. (αKG) α-ketoglutarate. Data are the mean of three biological replicates, and error bars represent s.e.m. $P < 0.05$ [*], $P < 0.005$ [***], Student's *t*-test. (c) Relative integrated peak intensities for pentose phosphate pathway-related metabolites. 6PG (6-phosphogluconic acid); R5P (ribose-5-phosphate); S7P (sedoheptulose-7-phosphate); E4P (erythrose-4-phosphate). Data are the mean of three biological replicates, and error bars represent s.e.m. $P < 0.05$ [*], $P < 0.005$ [***], Student's *t*-test. (d) Relative integrated peak intensities for purine biosynthetic precursors. R5P (ribose-5-phosphate); AICAR (5-amino-1-[3,4-dihydroxy-5-(hydroxymethyl)oxolan-2-yl]imidazole-4-carboxamide); IMP (inosine monophosphate); AMP (adenosine monophosphate); ADP (adenosine diphosphate); ATP (adenosine triphosphate). Data are the mean of three biological replicates, and error bars represent s.e.m. $P < 0.05$ [*], $P < 0.005$ [***], Student's *t*-test. (e) Relative integrated peak intensities for pyrimidine biosynthetic precursors. UMP (uridine monophosphate); UDP (uridine diphosphate); UTP (uridine triphosphate). Cells were treated with 25μM WQ-2101 for 24h. Integrated peak intensities were normalized to DMSO control. Data are the mean of three biological replicates, and error bars represent s.e.m. $P < 0.005$ [***], Student's *t*-test. (f) MTT assay of cells treated with DMSO control, 25μM WQ-2101, or 25μM WQ-2101 supplemented with reagents as indicated for 72h. OD540 values are relative to DMSO control. $P < 0.005$ [***], one-way ANOVA. (g) Representative image of cells upon nucleoside rescue. Scale bar = 100μm.

Supplementary Figure 4: PHGDH knockdown central carbon and nucleotide metabolism

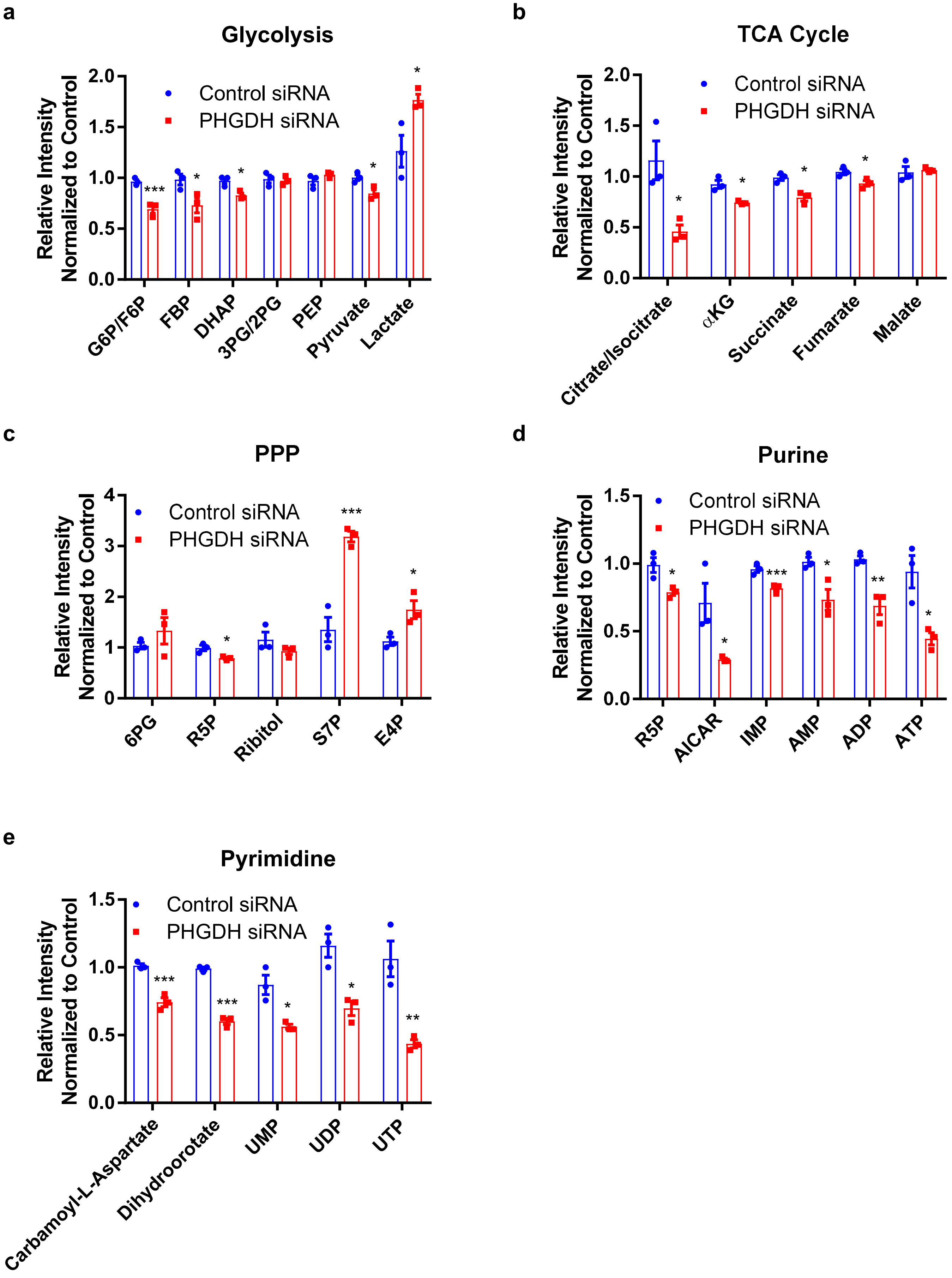

Supplementary Figure 4: PHGDH knockdown central carbon and nucleotide metabolism. (a) Relative integrated peak intensities for glycolysis-related metabolites. G6P/F6P (glucose-6-phosphate/fructose-6-phosphate); FBP (fructose-1,6-bisphosphate); DHAP (dihydroxyacetone-phosphate); 3PG/2PG (3-phosphoglycerate/2-phosphoglycerate); PEP (phosphoenolpyruvate). Data are the mean of three biological replicates, and error bars represent s.e.m. $P < 0.05$ [*], $P < 0.005$ [***], Student's *t*-test. (b) Relative integrated peak intensities for TCA cycle-related metabolites. (αKG) α-ketoglutarate. Data are the mean of three biological replicates, and error bars represent s.e.m. $P < 0.05$ [*], Student's *t*-test. (c) Relative integrated peak intensities for pentose phosphate pathway-related metabolites. 6PG (6-phosphogluconic acid); R5P (ribose-5-phosphate); S7P (sedoheptulose-7-phosphate); E4P (erythrose-4-phosphate). Data are the mean of three biological replicates, and error bars represent s.e.m. $P < 0.05$ [*], $P < 0.005$ [***], Student's *t*-test. (d) Relative integrated peak intensities for purine biosynthetic precursors. R5P (ribose-5-phosphate); AICAR (5-amino-1-[3,4-dihydroxy-5-(hydroxymethyl)oxolan-2-yl]imidazole-4-carboxamide); IMP (inosine monophosphate); AMP (adenosine monophosphate); ADP (adenosine diphosphate); ATP (adenosine triphosphate). Data are the mean of three biological replicates, and error bars represent s.e.m. $P < 0.05$ [*], $P < 0.01$ [**], $P < 0.005$ [***] Student's *t*-test. (e) Relative integrated peak intensities for pyrimidine biosynthetic precursors. UMP (uridine monophosphate); UDP (uridine diphosphate); UTP (uridine triphosphate). Data are the mean of three biological replicates, and error bars represent s.e.m. $P < 0.05$ [*], $P < 0.01$ [**], $P < 0.005$ [***], Student's *t*-test. For all panels, cells were transfected with 20 nM control siRNA or 20nM siRNA targeting *PHGDH* and assayed 72h post-transfection. Integrated peak intensities were normalized to control siRNA.

Supplementary Figure 5: Supplementary [U-^{13}C]Glucose labeling of nucleotides

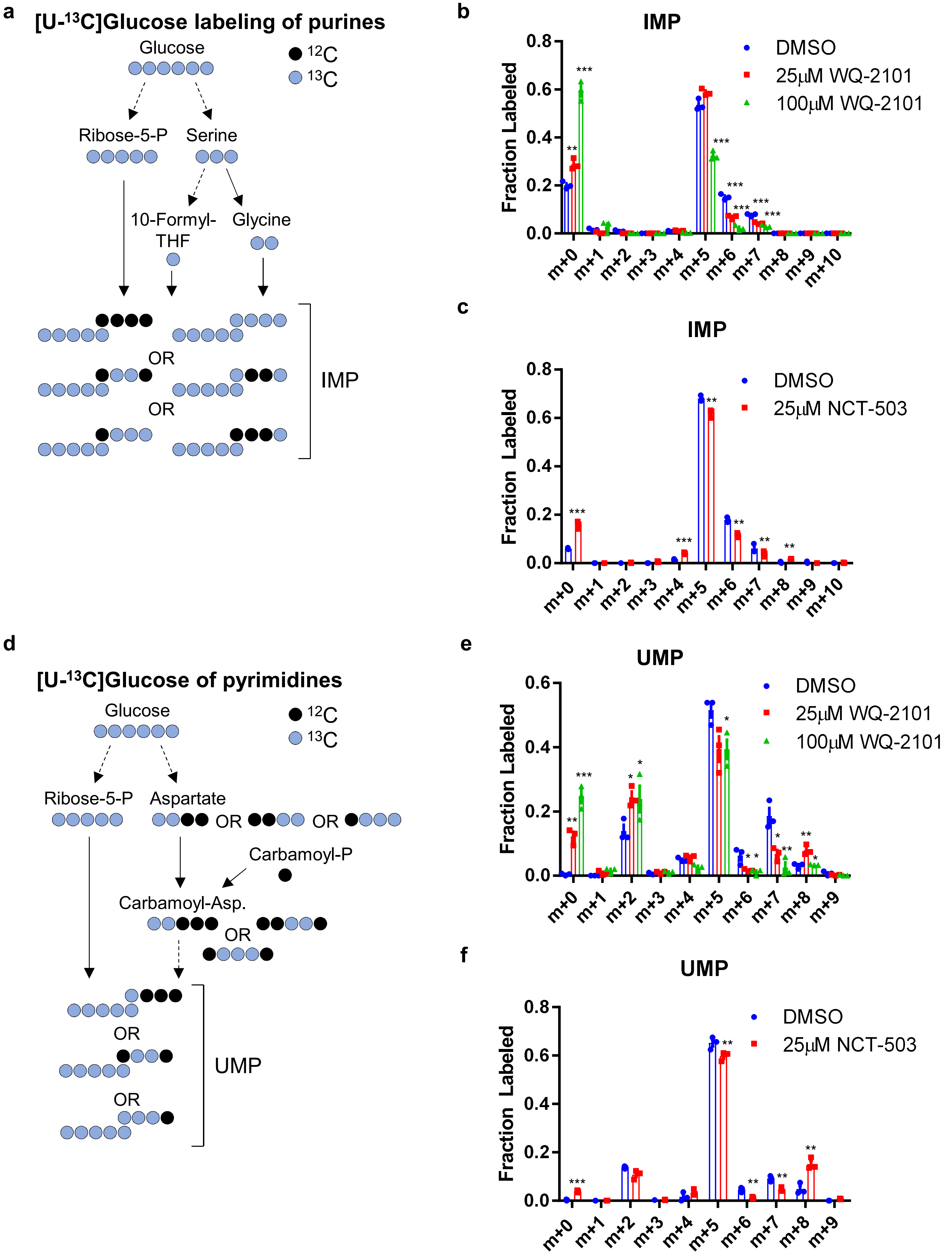

Supplementary Figure 5: Supplementary [U-^{13}C]Glucose labeling of nucleotides.
(a) Schematic of [U-^{13}C] glucose labeling of purines. (b,c) Mass-isotopomer distribution (MID) of IMP (inosine monophosphate) from [U-^{13}C] glucose in BT-20 cells treated with 25μM WQ-2101 (b) and HCT116 cells treated with 25μM NCT-503 (c). Data are the mean of three biological replicates, and error bars represent s.e.m. $P < 0.01$ [**], $P < 0.005$ [***], Student's *t*-test. (d) Schematic of [U-^{13}C] glucose labeling of pyrimidines. (e,f) Mass-isotopomer distribution (MID) of UMP (uridine monophosphate) from [U-^{13}C] glucose in BT-20 cells treated with 25μM WQ-2101 (e) and HCT116 cells treated with 25μM NCT-503 (f). Data are the mean of three biological replicates, and error bars represent s.e.m. $P < 0.05$ [*], $P < 0.01$ [**], $P < 0.005$ [***], Student's *t*-test. For labeling experiments, cells were pre-treated with inhibitors for 4h followed by introduction of [U-^{13}C] glucose-containing medium including inhibitors for 20h.

Supplementary Figure 6: WT and PHGDH KO [U-^{13}C]Glucose labeling of nucleotides

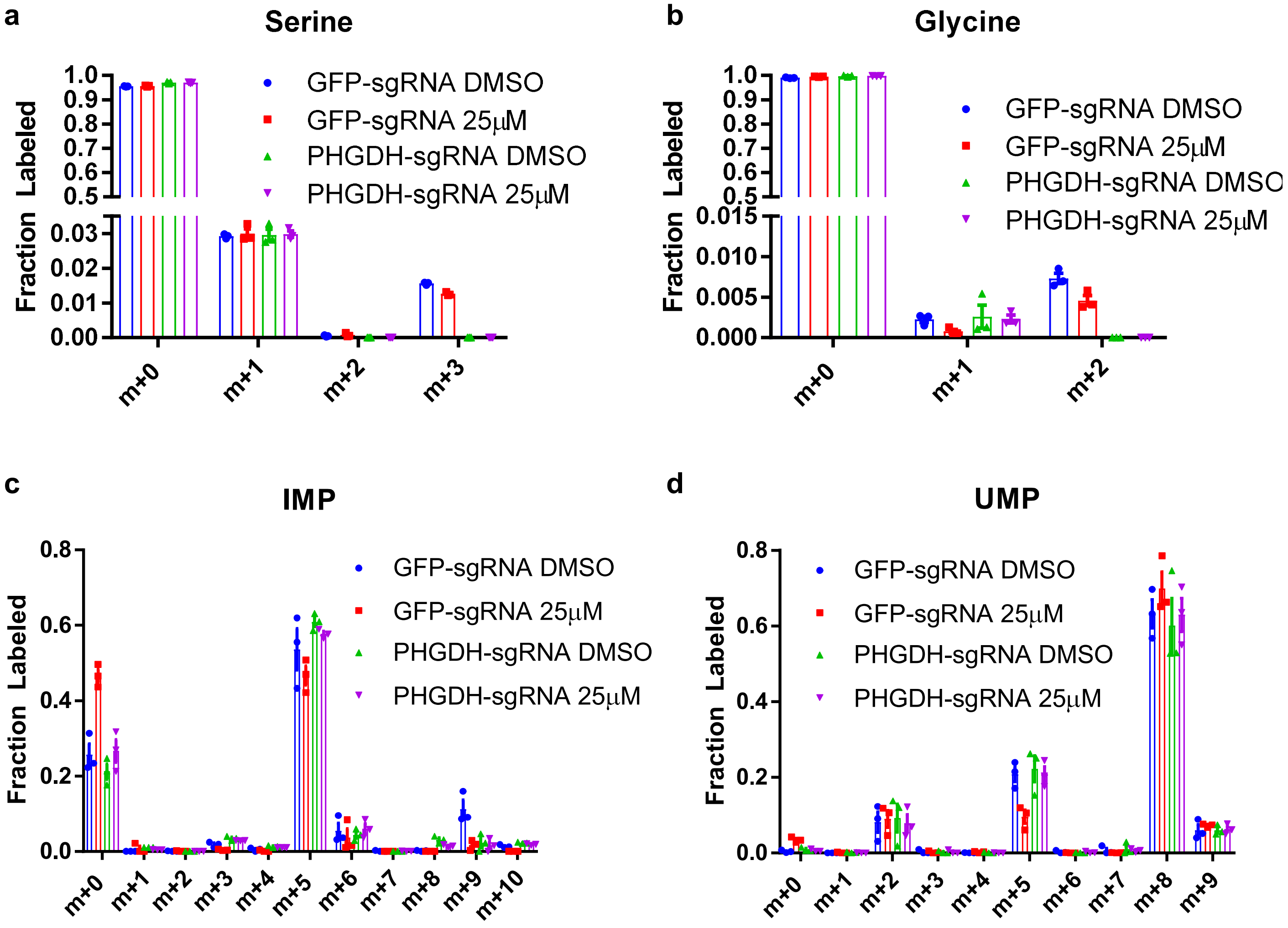

Supplementary Figure 6: WT and PHGDH KO [U-^{13}C]Glucose labeling of nucleotides. (a) Mass-isotopomer distribution (MID) of serine from [U-^{13}C] glucose in cells treated with 25µM WQ-2101. Data are the mean of three biological replicates, and error bars represent s.e.m. (b) Mass-isotopomer distribution (MID) of glycine from [U-^{13}C] glucose in cells treated with 25µM WQ-2101. Data are the mean of three biological replicates, and error bars represent s.e.m (c) Mass-isotopomer distribution (MID) of IMP (inosine monophosphate) from [U-^{13}C] glucose in cells treated with 25µM WQ-2101. Data are the mean of three biological replicates, and error bars represent s.e.m. (d) Mass-isotopomer distribution (MID) of UMP (uridine monophosphate) from [U-^{13}C] glucose in cells treated with 25µM WQ-2101. Data are the mean of three biological replicates, and error bars represent s.e.m. For labeling experiments, cells were pre-treated with inhibitors for 4h followed by introduction of [U-^{13}C] glucose-containing medium including inhibitors for 20h.

Supplementary Figure 7: Supplementary Metabolic flux analysis

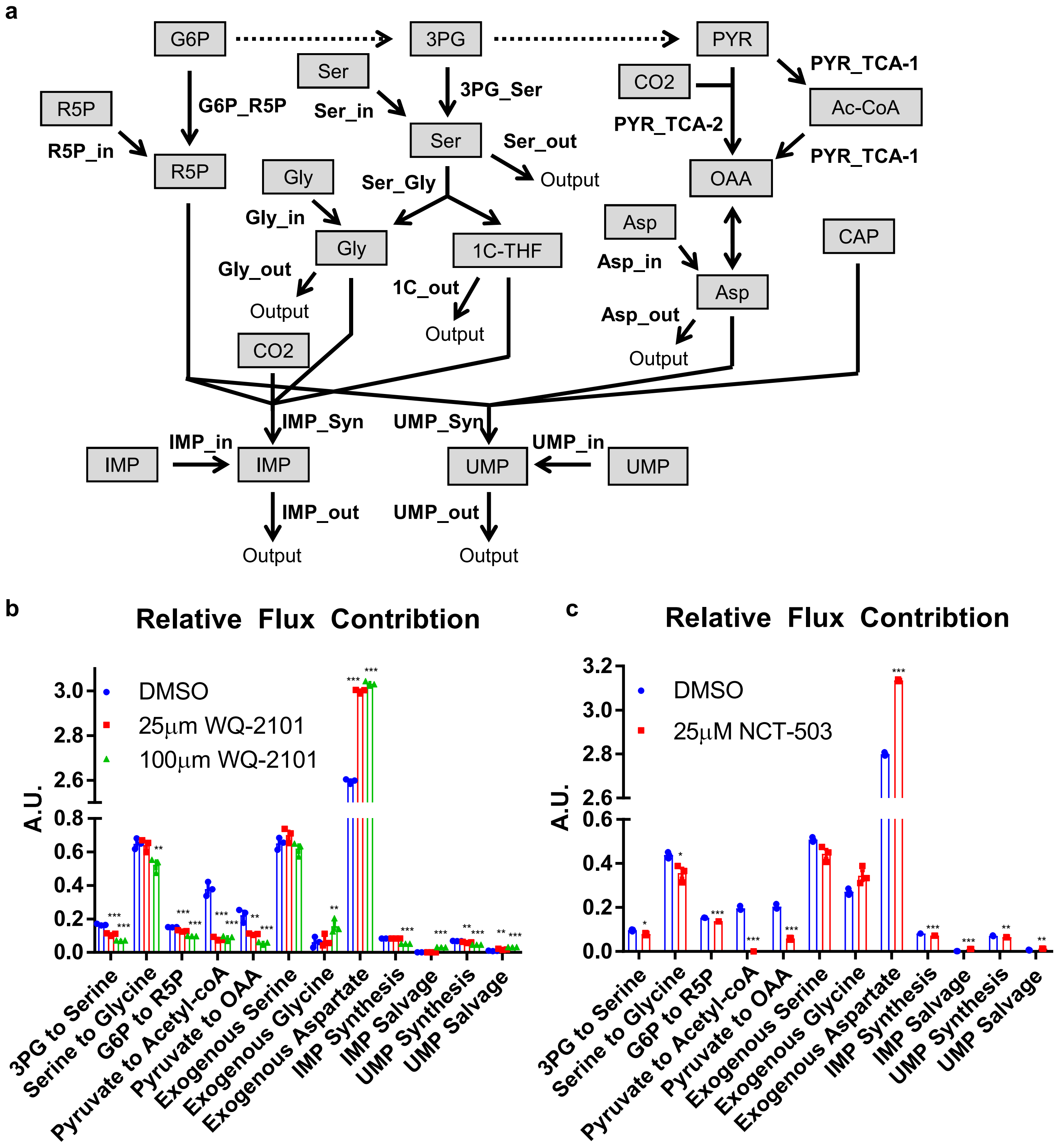

Supplementary Figure 7: Supplementary metabolic flux analysis.
(a) Schematic of metabolic flux analysis model. R5P (ribose-5-phosphate); G6P (glucose-6-phosphate); Ser (serine); Gly (glycine), 3PG (3-phosphoglycerate); CO_2 (carbon dioxide); PYR (pyruvate); Ac-CoA (acetyl-coA); OAA (oxaloacetate); Asp (aspartate); CAP (carbamoyl phosphate); UMP (uridine monophosphate); IMP (inosine monophosphate). (b,c) Relative flux rate of metabolic pathways in BT-20 cells treated with 25μM WQ-2101 or 100 μM WQ-2101 (b) and HCT116 cells treated with 25μM NCT-503 (c). Data are the mean of three biological replicates, and error bars represent s.e.m. $P < 0.05$ [*], $P < 0.01$ [**], $P < 0.005$ [***], Student's *t*-test.

Supplementary Figure 8: BT-20 kinetic flux profiling

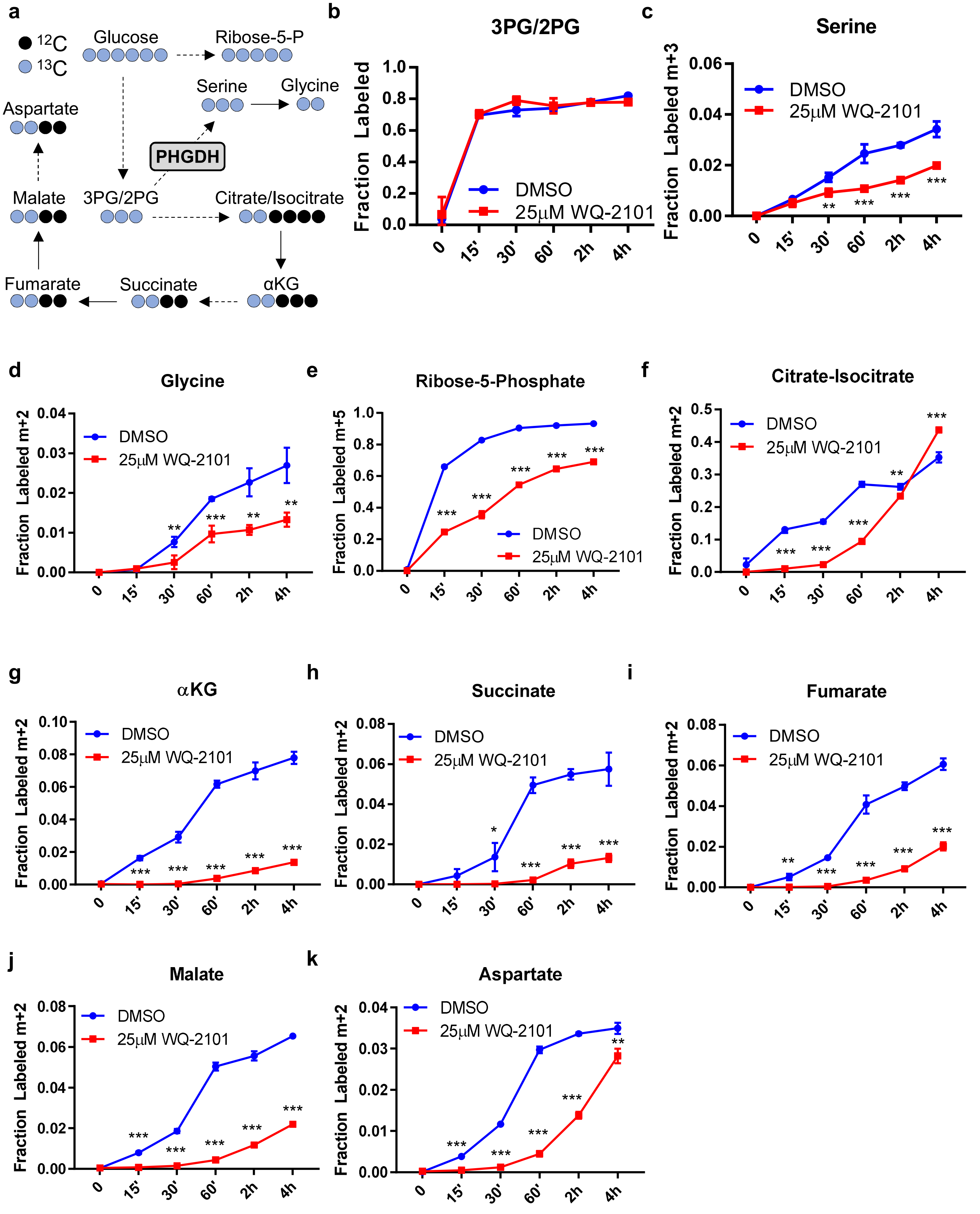

Supplementary Figure 8: BT-20 kinetic flux profiling.

(a) Schematic of [U-^{13}C] glucose labeling of serine, glycine, and central carbon metabolites. (b) Fraction of the m+3 isotopomer of 3PG/2PG (3-phosphoglycerate/2-phosphoglycerate) labeled in the presence (red) or absence (blue) of 25μM WQ-2101. 3PG/2PG (3-phosphoglycerate/2-phosphoglycerate0). Data are the mean of three biological replicates, and error bars represent s.e.m. (c) Fraction of the m+3 isotopomer of serine labeled in the presence (red) or absence (blue) of 25μM WQ-2101. Data are the mean of three biological replicates, and error bars represent s.e.m. $P < 0.01$ [**], $P < 0.005$ [***], Student's *t*-test. (d) Fraction of the m+2 isotopomer of glycine labeled in the presence (red) or absence (blue) of 25μM WQ-2101. Data are the mean of three biological replicates, and error bars represent s.e.m. $P < 0.01$ [**], $P < 0.005$ [***], Student's *t*-test. (e) Fraction of the m+5 isotopomer of ribose-5-phosphate labeled in the presence (red) or absence (blue) of 25μM WQ-2101. Data are the mean of three biological replicates, and error bars represent s.e.m. $P < 0.005$ [***], Student's *t*-test. (f-k) Fraction of the m+2 isotopomers of citrate/isocitrate, αKG (α-ketoglutarate), succinate, fumarate, malate, and aspartate labeled in the presence (red) or absence (blue) of 25μM WQ-2101. Data are the mean of three biological replicates, and error bars represent s.e.m. $P < 0.05$ [*], $P < 0.01$ [**], $P < 0.005$ [***], Student's *t*-test. In all experiments, cells were pre-treated with 25μM WQ-2101 for 24h followed by introduction of [U-^{13}C] glucose-containing medium including inhibitor for the indicated times.

Supplementary Figure 9: NCT-503 kinetic flux profiling

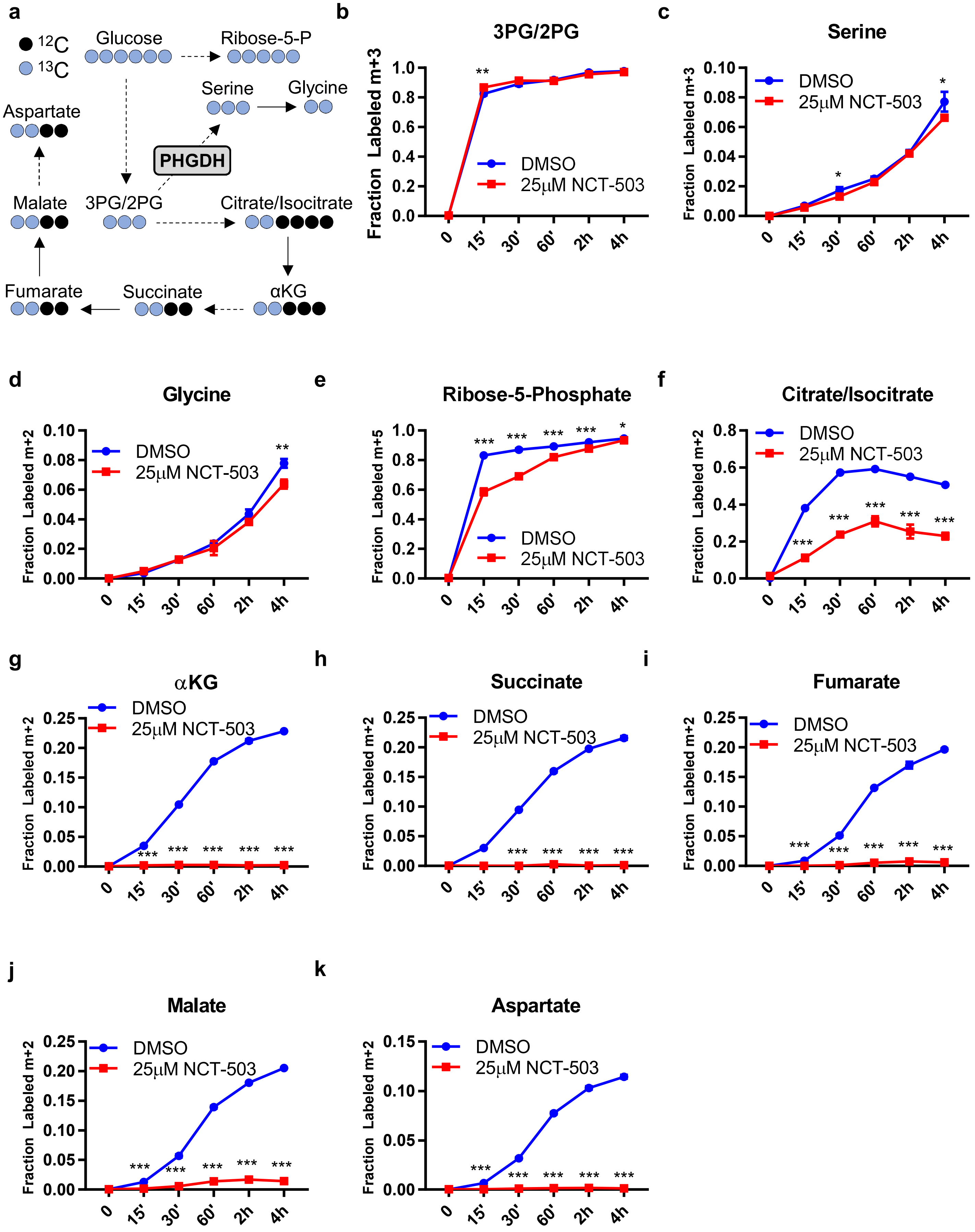

Supplementary Figure 9: NCT-503 kinetic flux profiling.
(a) Schematic of [U-^{13}C] glucose labeling of serine, glycine, and central carbon metabolites. (b) Fraction of the m+3 isotopomer of 3PG/2PG (3-phosphoglycerate/2-phosphoglycerate) labeled in the presence (red) or absence (blue) of 25μM NCT-503. 3PG/2PG (3-phosphoglycerate/2-phosphoglycerate). Data are the mean of three biological replicates, and error bars represent s.e.m. $P < 0.01$ [**], Student's *t*-test. (c) Fraction of the m+3 isotopomer of serine labeled in the presence `(red) or absence (blue) of 25μM NCT-503. Data are the mean of three biological replicates, and error bars represent s.e.m. $P < 0.05$ [*], Student's *t*-test. (d) Fraction of the m+2 isotopomer of glycine labeled in the presence (red) or absence (blue) of 25μM NCT-503. Data are the mean of three biological replicates, and error bars represent s.e.m. $P < 0.01$ [**], Student's *t*-test. (e) Fraction of the m+5 isotopomer of ribose-5-phosphate labeled in the presence (red) or absence (blue) of 25μM NCT-503. Data are the mean of three biological replicates, and error bars represent s.e.m. $P < 0.05$ [*], $P < 0.005$ [***], Student's *t*-test. (f-k) Fraction of the m+2 isotopomers of citrate/isocitrate, αKG (α-ketoglutarate), succinate, fumarate, malate, and aspartate labeled in the presence (red) or absence (blue) of 25μM NCT-503. Data are the mean of three biological replicates, and error bars represent s.e.m. $P < 0.005$ [***], Student's *t*-test. In all experiments, cells were pre-treated with 25μM NCT-503 for 24h followed by introduction of [U-^{13}C] glucose-containing medium including inhibitor for the indicated times.

Supplementary Figure 10: [U-^{13}C]Glutamine labeling of TCA cycle

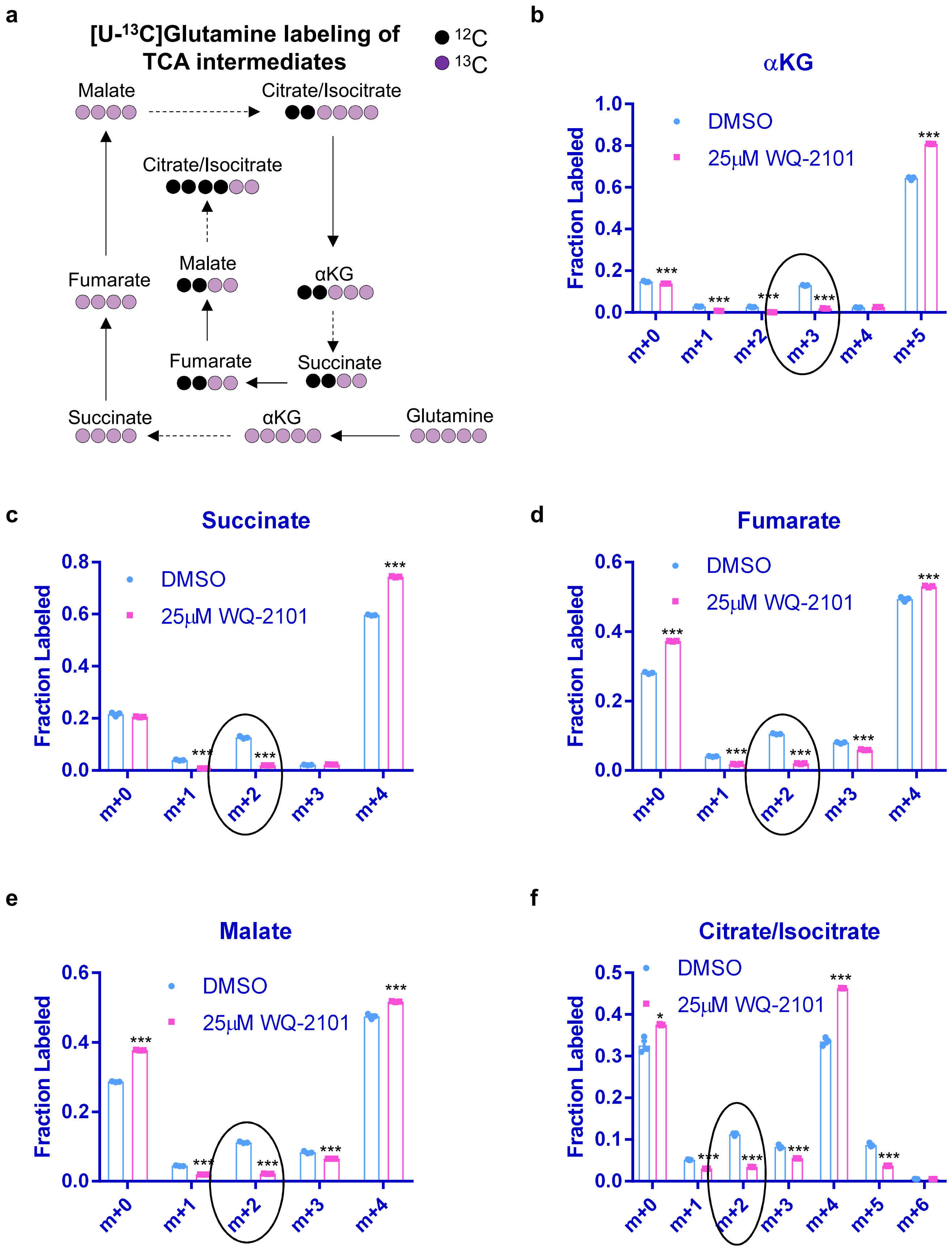

Supplementary Figure 10: [U-^{13}C]Glutamine labeling of TCA cycle.

(a) Schematic of [U-^{13}C] glutamine labeling of TCA cycle intermediates. (b) Mass-isotopomer distribution (MID) of αKG (α-ketoglutarate) from [U-^{13}C] glutamine in cells treated with DMSO control or 25μM WQ-2101. Data are the mean of three biological replicates, and error bars represent s.e.m. $P < 0.005$ [***], Student's *t*-test. (c) MID of succinate from [U-^{13}C] glutamine in cells treated with DMSO control or 25μM WQ-2101. Data are the mean of three biological replicates, and error bars represent s.e.m. $P < 0.005$ [***], Student's *t*-test. (d) MID of fumarate from [U-^{13}C] glutamine in cells treated with DMSO control or 25μM WQ-2101. Data are the mean of three biological replicates, and error bars represent s.e.m. $P < 0.005$ [***], Student's *t*-test. (e) MID of malate from [U-^{13}C] glutamine in cells treated with DMSO control or 25μM WQ-2101. Data are the mean of three biological replicates, and error bars represent s.e.m. $P < 0.005$ [***], Student's *t*-test. (f) MID of citrate/isocitrate from [U-^{13}C] glutamine in cells treated with DMSO control or 25μM WQ-2101. Data are the mean of three biological replicates, and error bars represent s.e.m. $P < 0.05$ [*], $P < 0.005$ [***], Student's *t*-test. In all experiments, cells were pre-treated with DMSO or 25μM WQ-2101 for 4h followed by introduction of [U-^{13}C] glucose-containing medium including DMSO or WQ-2101 for 20h.

Supplementary Figure 11: WT and PHGDH KO kinetic flux profiling

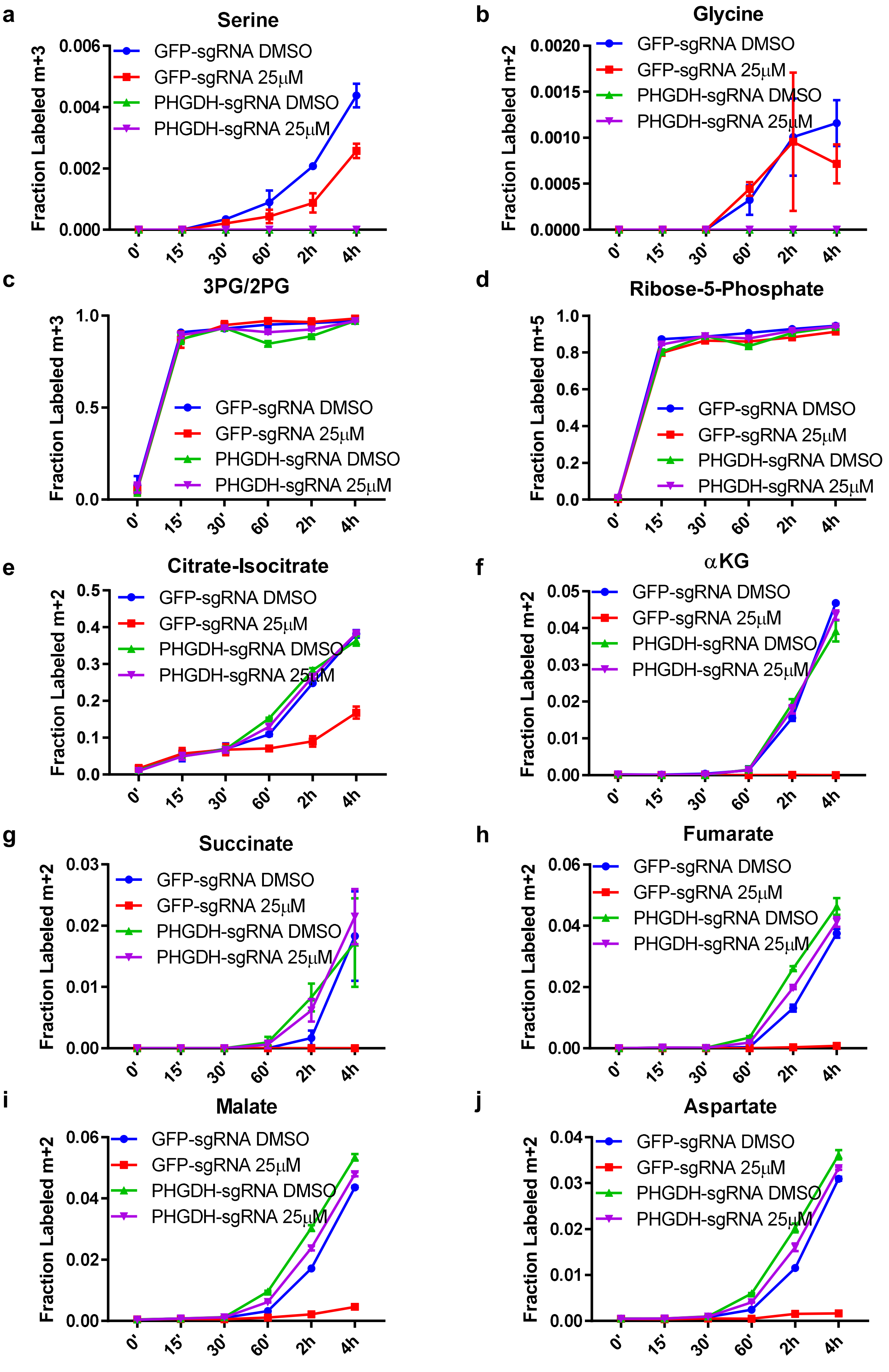

Supplementary Figure 11: WT and PHGDH KO kinetic flux profiling.
(a) Fraction of the m+3 isotopomer of serine labeled in the presence or absence of 25μM WQ-2101 in wildtype or PHGDH knockout cells. Data are the mean of three biological replicates, and error bars represent s.e.m. (b) Fraction of the m+2 isotopomer of glycine labeled in the presence or absence of 25μM WQ-2101 in wildtype or PHGDH knockout cells. Data are the mean of three biological replicates, and error bars represent s.e.m. (c) Fraction of the m+3 isotopomer of 3PG/2PG (3-phosphoglycerate/2-phosphoglycerate) labeled in the presence or absence of 25μM WQ-2101 in wildtype or PHGDH knockout cells. 3PG/2PG (3-phosphoglycerate/2-phosphoglycerate). Data are the mean of three biological replicates, and error bars represent s.e.m (d) Fraction of the m+5 isotopomer of ribose-5-phosphate labeled in the presence or absence of 25μM WQ-2101 in wildtype or PHGDH knockout cells. Data are the mean of three biological replicates, and error bars represent s.e.m. (e-j) Fraction of the m+2 isotopomers of citrate/isocitrate, αKG (α-ketoglutarate), succinate, fumarate, malate, and aspartate labeled the presence or absence of 25μM WQ-2101 in wildtype or PHGDH knockout cells. Data are the mean of three biological replicates, and error bars represent s.e.m. In all experiments, cells were pre-treated with 25μM WQ-2101 for 24h followed by introduction of [U-^{13}C] glucose-containing medium including inhibitor for the indicated times.

Supplementary Figure 12: PHGDH siRNA and NCT-503 TCA cycle and pentose phosphate pathway rescue

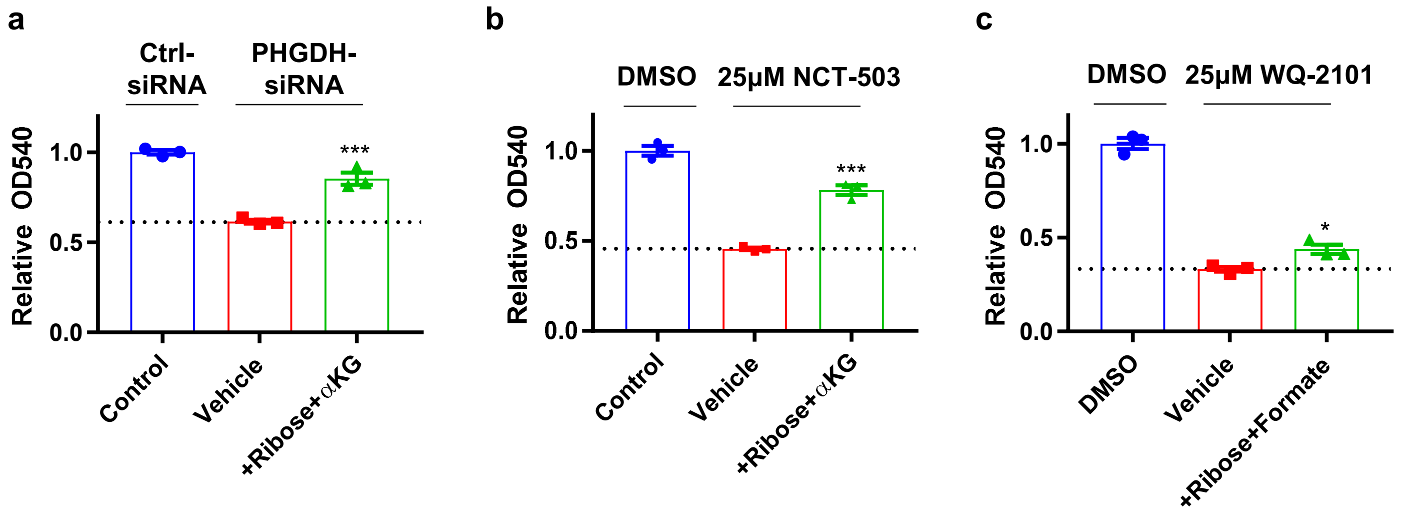

Supplementary Figure 12: PHGDH siRNA and NCT-503 TCA cycle and pentose phosphate pathway rescue.

(a) MTT assay of cells transfected with 20nM control siRNA or 20nM PHGDH siRNA for 48h followed by supplementation with ribose (15mM) and di-methyl α-ketoglutarate (αKG, 3mM) for 72h. OD540 values are relative to DMSO control. $P < 0.005$ [***], one-way ANOVA. (b) MTT assay of cells treated with DMSO control, 25μM WQ-2101, or 25μM WQ-2101 supplemented with ribose (15mM) and di-methyl α-ketoglutarate (αKG, 3mM) for 72h. OD540 values are relative to DMSO control. $P < 0.005$ [***], one-way ANOVA. (c) MTT assay of cells treated with DMSO control, 25μM WQ-2101, or 25μM WQ-2101 supplemented with ribose (15mM) and formate (0.5mM) for 72h. OD540 values are relative to DMSO control. $P < 0.05$ [*], one-way ANOVA.

Supplementary Figure 13: Uncropped western blots

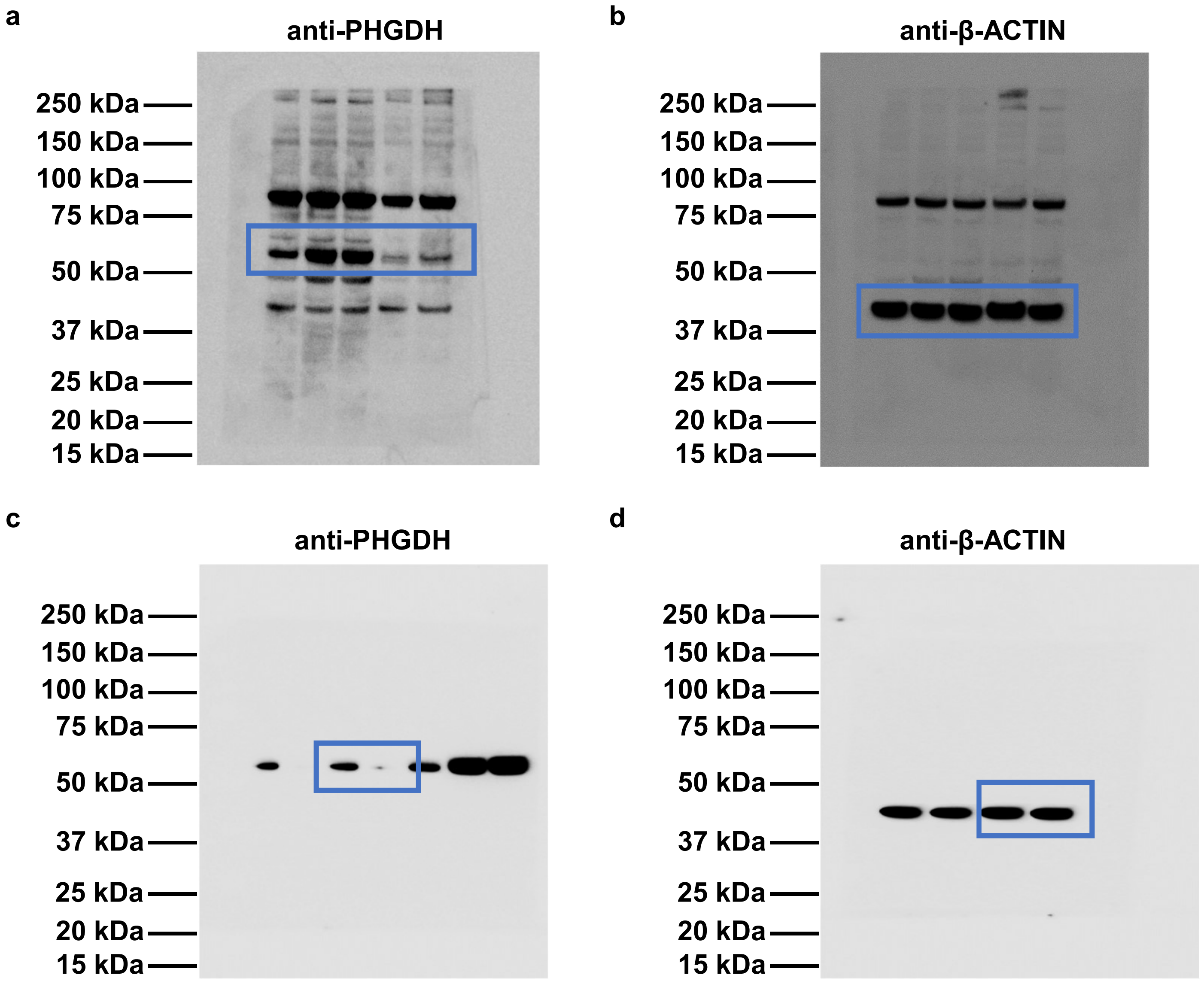

Supplementary Figure 13: Uncropped western blots.
(a) anti-PHGDH western blot from Figure 1c. (b) anti-β-ACTIN western blot from Figure 1c. (c) anti-PHGDH western blot from Supplementary Figure 1b. (d) anti-β-ACTIN western blot from Supplementary Figure 1b. Blue square indicates portion displayed in respective figures.

Supplementary Note 1: Construction of the metabolic flux analysis model

Metabolic network model:

A metabolic network model (Table 1) including the TCA cycle, serine/glycine/one carbon metabolism, oxidative pentose phosphate pathway and nucleotide synthesis was used in the metabolic flux analysis. For simplicity, the TCA cycle is represented as the PYR_TCA-1 and PYR_TCA-2 reactions, in which carbon atoms from pyruvate feed the TCA cycle through reactions catalyzed by PDH and PC. For the metabolites ribose-5-phosphate (R5P), serine (Ser), glycine (Gly), aspartate (Asp), inosine monophosphate (IMP) and uridine monophosphate (UMP), contribution from unlabeled sources such as the salvage pathways were also considered in this model. Coefficients in the biomass synthesis flux were evaluated from the molecular composition of dry cell weight from literature[1] (Table 2).

Table 1. Reactions and carbon atom mapping relationships in the model

Reaction	Stoichiometry and carbon atom mapping
Enzyme catalyzed reactions	
G6P_R5P (G6PD)	G6P (abcdef) → R5P (bcdef) + CO2 (a)
3PG_Ser (PHGDH)	3PG (abc) → Ser (abc)
Ser_Gly (SHMT)	Ser (abc) → Gly (ab) + 1C-THF (c)
PYR_TCA-1 (PDH)	PYR (abc) → Ac-CoA (bc) + CO2 (a)<br>Ac-CoA (ab) + Ac-CoA (cd) + Ac-CoA (ef) → OAA (abdf) + CO2 (c) + CO2 (e)
PYR_TCA-2 (PC)	PYR (abc) + CO2 (d) → OAA (abcd)
OAA_Asp (AST)	OAA (abcd) → Asp (abcd)
IMP_Syn	R5P (abcde) + Gly (fg) + CO2 (h) + 1CTHF (i) + 1CTHF (j) → IMP (abcdefghij)
UMP_Syn	R5P (abcde) + Asp (fghi) + CAP (j) → UMP (abcdeghij) + CO2 (f)
Input fluxes from unlabeled sources	
R5P_In	R5P_input (abcde) → R5P (abcde)
Ser_In	Ser_input (abc) → Ser (abc)
Gly_In	Gly_input (ab) → Gly (ab)
Asp_In	Asp_input (abcd) → Asp (abcd)
IMP_In	IMP_input (abcdefghij) → IMP (abcdefghij)
UMP_In	UMP_input (abcdefghi) → UMP (abcdefghi)
Biomass synthesis	
Biomass	0.63 Gly + 0.17 Ser + 3.13 Asp + 0.083 IMP + 0.078 UMP →Biomass

Table 2. Literature values of precursor coefficients in biomass synthesis flux

Biomass precursor	Coefficient
Glycine	0.63

Serine	0.17
Aspartate	3.13
AMP	0.02
CMP	0.04
GMP	0.04
UMP	0.02
dAMP	0.01
dCMP	0.01
dGMP	0.01
dTMP	0.01

Since the biomass precursors AMP, GMP, dAMP, dGMP, CMP, dCMP and dTMP are not explicitly included in our model, their contribution to biomass synthesis is conferred by their precursors IMP and UMP, respectively. Thus, the coefficient for a biomass precursor in our model can be estimated by adding up the coefficients for all metabolites in Table 2 that can be derived from this precursor. These coefficients are listed in Table 3.

Table 3. Coefficients of precursors in biomass synthesis flux in model

Biomass precursor	Related metabolite in Table 2	Coefficient
Glycine	Glycine	0.63
Serine	Serine	0.17
Aspartate	Aspartate	3.13
IMP	AMP + GMP + dAMP + dGMP	0.08
UMP	CMP + UMP + dCMP + dTMP	0.08

Elementary metabolite unit (EMU) decomposition of the model

An EMU model including the TCA cycle, serine/glycine/one carbon metabolism, pentose phosphate pathway and nucleotide synthesis was constructed based on the stoichiometric matrix and carbon atom mapping in Table 1. The resulting model is described by the following matrix equations:

$$[-F_{\mathrm{IMP\_Syn}} - F_{\mathrm{IMP\_in}}][\mathrm{IMP}_{1111111111}] = [-F_{\mathrm{IMP\_Syn}} \quad -F_{\mathrm{IMP\_in}}]\begin{bmatrix}\mathrm{R5P}_{11111} \times \mathrm{Gly}_{11} \times \mathrm{CO2}_1 \times \mathrm{1CTHF}_1 \times \mathrm{1CTHF}_1 \\ \mathrm{IMP\_input}_{1111111111}\end{bmatrix} \quad (1)$$

$$[-F_{\mathrm{UMP\_Syn}} - F_{\mathrm{UMP\_in}}][\mathrm{UMP}_{111111111}] = [-F_{\mathrm{UMP\_Syn}} \quad -F_{\mathrm{UMP\_in}}]\begin{bmatrix}\mathrm{R5P}_{11111} \times \mathrm{Asp}_{0111} \times \mathrm{CAP}_1 \\ \mathrm{UMP\_input}_{111111111}\end{bmatrix} \quad (2)$$

$$[-F_{\text{R5P\_Syn}} - F_{\text{R5P\_in}}][\text{R5P}_{11111}] = [-F_{\text{G6P\_R5P}} \quad -F_{\text{R5P\_in}}]\begin{bmatrix}\text{G6P}_{011111}\\ \text{R5P\_input}_{11111}\end{bmatrix} \tag{3}$$

$$\begin{bmatrix} F_{\text{OAA\_Asp}} & -F_{\text{OAA\_Asp}} - F_{\text{Asp\_in}} \\ -F_{\text{PYR\_TCA\_1}} - F_{\text{PYR\_TCA\_2}} & 0 \end{bmatrix}\begin{bmatrix}\text{OAA}_{1111}\\ \text{Asp}_{1111}\end{bmatrix} =$$

$$\begin{bmatrix} 0 & 0 & -F_{\text{Asp\_in}} \\ -F_{\text{PYR\_TCA\_1}} & -F_{\text{PYR\_TCA\_2}} & 0 \end{bmatrix}\begin{bmatrix}\text{PYR}_{011} \times \text{PYR}_{001} \times \text{PYR}_{001}\\ \text{PYR}_{111} \times \text{CO2}_{1} \\ \text{Asp\_input}_{1111}\end{bmatrix} \tag{4}$$

$$\begin{bmatrix} F_{\text{OAA\_Asp}} & -F_{\text{OAA\_Asp}} - F_{\text{Asp\_in}} & 0 \\ -F_{\text{PYR\_TCA\_1}} - F_{\text{PYR\_TCA\_2}} & 0 & 0 \\ 0 & 0 & -F_{\text{3PG\_Ser}} - F_{\text{Ser\_in}} \end{bmatrix}\begin{bmatrix}\text{OAA}_{0111}\\ \text{Asp}_{0111} \\ \text{Ser}_{111}\end{bmatrix} =$$

$$\begin{bmatrix} 0 & 0 & -F_{\text{Asp\_in}} & 0 & 0 \\ -F_{\text{PYR\_TCA\_1}} & -F_{\text{PYR\_TCA\_2}} & 0 & 0 & 0 \\ 0 & 0 & 0 & -F_{\text{3PG\_Ser}} & -F_{\text{Ser\_in}} \end{bmatrix}\begin{bmatrix}\text{PYR}_{001} \times \text{PYR}_{001} \times \text{PYR}_{001}\\ \text{PYR}_{011} \times \text{CO2}_{1} \\ \text{Asp\_input}_{0111} \\ \text{3PG}_{111} \\ \text{Ser\_input}_{111}\end{bmatrix} \tag{5}$$

$$\begin{bmatrix} -F_{\text{3PG\_Ser}} - F_{\text{Ser\_in}} & 0 \\ F_{\text{Ser\_Gly}} & -F_{\text{Ser\_Gly}} - F_{\text{Gly\_in}} \end{bmatrix}\begin{bmatrix}\text{Ser}_{110}\\ \text{Gly}_{11}\end{bmatrix} = \begin{bmatrix} -F_{\text{3PG\_Ser}} & -F_{\text{Ser\_in}} & 0 \\ 0 & 0 & -F_{\text{Gly\_in}} \end{bmatrix}\begin{bmatrix}\text{3PG}_{110}\\ \text{Ser\_input}_{110} \\ \text{Gly\_input}_{11}\end{bmatrix} \tag{6}$$

$$[-F_{\text{3PG\_Ser}} - F_{\text{Ser\_in}}][\text{1CTHF}_{1}] = [-F_{\text{3PG\_Ser}} \quad -F_{\text{Ser\_in}}]\begin{bmatrix}\text{3PG}_{001}\\ \text{Ser\_input}_{001}\end{bmatrix} \tag{7}$$

The subscript of each EMU refers to the carbon atoms included in that EMU. For example, Asp_{0111} is the EMU with the 2nd, 3rd and 4th carbon atoms in aspartate. EMUs with the suffix 'input' refer to EMUs in unlabeled sources (e.g. salvage pathways) that also contribute to the production of the corresponding metabolite. F denotes the metabolic flux through a reaction. Experimentally measured MIDs of IMP, UMP, G6P, R5P, PYR, OAA/Asp, 3PG, Ser and Gly were used in estimating the metabolic fluxes. CAP, CO_2 and all 'input' EMUs were assumed to be unlabeled, in which each carbon atom has the probability 0.01109 to contain ^{13}C according to the natural isotope abundance. MIDs of Asp_{0111}, G6P_{011111}, PYR_{011}, PYR_{001}, Asp_{0011}, Ser_{110} and 3PG_{110} were estimated from the experimentally measured MIDs of the metabolites directly containing these EMUs based on the assumption that each

carbon atom has the same probability to be labeled. The MID of 1CTHF was estimated from the MIDs of 3PG and Ser_input based on the same assumption.

Solving for the relative metabolic fluxes from the EMU model

The EMU model was solved in two steps to determine the metabolic fluxes from mass isotopomer distributions (MIDs). In the first step, flux ratios at branch points were directly computed from MIDs of the EMUs linked to the respective branch point. The flux ratios were then used in combination with the stoichiometric matrix to solve all relative metabolic fluxes in the model.

Step 1. Calculate flux ratios at branch points:

For a given branch point with two upstream fluxes F_1 and F_2, suppose that $\mathbf{M}_1$ and $\mathbf{M}_2$ and are the MID vectors calculated from the convolution of MIDs of all EMUs directly converted to the related branch point metabolite by F_1 and F_2, respectively, and $\mathbf{M}_3$ is MID of the metabolite at the branch point (i.e. $\mathbf{M}_3$ can be derived from either $\mathbf{M}_1$ or $\mathbf{M}_2$), then the isotopomer balance equations for this branch point are:

$$F_1\mathbf{M}_1 + F_2\mathbf{M}_2 - (F_1 + F_2)\mathbf{M}_3 = 0 \tag{8}$$

Let $R = \frac{F_1}{F_1+F_2}$ be the flux ratio at this branch point, we have:

$$R\mathbf{M}_1 + (1 - R)\mathbf{M}_2 = \mathbf{M}_3 \tag{9}$$

Which is identical to:

$$(\mathbf{M}_1 - \mathbf{M}_2)R = \mathbf{M}_3 - \mathbf{M}_2 \tag{10}$$

Because the dimension of the vector $(n + 1)$ is always larger than 1, the equations above are overdetermined. The equations were therefore solved using by least-squares minimization. This was implemented using the function numpy.linalg.lstsq in the Python package NumPy.

We note that most branch points in our model consist of one input flux from an unlabeled source and a flux synthesizing this metabolite from its precursor. Metabolites at these branch points include IMP, UMP, R5P, Asp, Ser and Gly. Thus, the equations for the corresponding flux ratios are:

$$R_{\text{IMP\_in}}\text{IMP\_input}_{1111111111} + (1 - R_{\text{IMP\_in}})(\text{R5P}_{11111} \times \text{Gly}_{11} \times \text{CO2}_1 \times \text{1CTHF}_1 \times \text{1CTHF}_1) = \text{IMP}_{1111111111} \quad (11)$$

$$R_{\text{UMP\_in}}\text{UMP\_input}_{111111111} + (1 - R_{\text{UMP\_in}})(\text{R5P}_{11111} \times \text{Asp}_{0111} \times \text{CAP}_1) = \text{UMP}_{111111111} \quad (12)$$

$$R_{\text{R5P\_in}}\text{R5P\_input}_{11111} + (1 - R_{\text{R5P\_in}})\text{G6P}_{011111} = \text{R5P}_{11111} \quad (13)$$

$$R_{\text{Asp\_in}}\text{Asp\_input}_{1111} + R_{\text{Asp\_TCA\_2}}(\text{PYR}_{111} \times \text{CO2}_1) + (1 - R_{\text{Asp\_in}} - R_{\text{Asp\_TCA\_2}})(\text{PYR}_{011} \times \text{PYR}_{001} \times \text{PYR}_{001}) = \text{Asp}_{1111} \quad (14)$$

$$R_{\text{Asp\_in}}\text{Asp\_input}_{0111} + R_{\text{Asp\_TCA\_2}}(\text{PYR}_{011} \times \text{CO2}_1) + (1 - R_{\text{Asp\_in}} - R_{\text{Asp\_TCA\_2}})(\text{PYR}_{001} \times \text{PYR}_{001} \times \text{PYR}_{001}) = \text{Asp}_{0111} \quad (15)$$

$$R_{\text{Ser\_in}}\text{Ser\_input}_{111} + (1 - R_{\text{Ser\_in}})\text{3PG}_{111} = \text{Ser}_{111} \quad (16)$$

$$R_{\text{Gly\_in}}\text{Gly\_input}_{11} + (1 - R_{\text{Gly\_in}})\text{Ser}_{110} = \text{Gly}_{11} \quad (17)$$

$$R_{\text{Ser\_in}}\text{Ser\_input}_{001} + (1 - R_{\text{Ser\_in}})\text{3PG}_{001} = \text{1CTHF}_1 \quad (18)$$

Step 2. Calculate relative fluxes from flux ratios and stoichiometric matrix:

Given the flux ratios estimated in the previous step, all relative fluxes can therefore be determined by solving the equations below:

$$F_{\text{Gly\_out}}:F_{\text{Ser\_out}}:F_{\text{Asp\_out}}:F_{\text{IMP\_out}}:F_{\text{UMP\_out}} = 0.63:0.17:3.13:0.083:0.078 \quad (19)$$

$$F_{\text{IMP\_Syn}} = (1 - R_{\text{IMP\_in}})F_{\text{IMP\_out}} \quad (20)$$

$$F_{\text{IMP\_in}} = R_{\text{IMP\_in}}F_{\text{IMP\_out}} \quad (21)$$

$$F_{\text{UMP\_Syn}} = (1 - R_{\text{UMP\_in}})F_{\text{UMP\_out}} \quad (22)$$

$$F_{\text{UMP\_in}} = R_{\text{IMP\_in}}F_{\text{UMP\_out}} \quad (23)$$

$$F_{\text{Ser\_Gly}} = (1 - R_{\text{Gly\_in}})(F_{\text{Gly\_out}} + F_{\text{IMP\_Syn}}) \quad (24)$$

$$F_{\text{Gly\_in}} = R_{\text{Gly\_in}}(F_{\text{Gly\_out}} + F_{\text{IMP\_Syn}}) \quad (25)$$

$$F_{\text{3PG\_Ser}} = (1 - R_{\text{Ser\_in}})(F_{\text{Ser\_out}} + F_{\text{Ser\_Gly}}) \quad (26)$$

$$F_{\text{Ser\_in}} = R_{\text{Ser\_in}}(F_{\text{Ser\_out}} + F_{\text{Ser\_Gly}}) \quad (27)$$

$$F_{\text{G6P\_R5P}} = (1 - R_{\text{R5P\_in}})(F_{\text{IMP\_Syn}} + F_{\text{UMP\_Syn}}) \quad (28)$$

$$F_{\text{PYR\_TCA\_1}} = (1 - R_{\text{Asp\_TCA\_2}} - R_{\text{Asp\_in}})(F_{\text{Asp\_out}} + F_{\text{UMP\_Syn}}) \quad (29)$$

$$F_{\text{Asp\_TCA\_2}} = R_{\text{Asp\_TCA\_2}}(F_{\text{Asp\_out}} + F_{\text{UMP\_Syn}}) \quad (30)$$

$$F_{\text{Asp\_in}} = R_{\text{Asp\_in}}(F_{\text{Asp\_out}} + F_{\text{UMP\_Syn}}) \quad (31)$$

Supplementary References

1. Shlomi, T., Benyamini, T., Gottlieb, E., Sharan, R. & Ruppin, E. Genome-scale metabolic modeling elucidates the role of proliferative adaptation in causing the Warburg effect. PLoS Comput Biol 7, e1002018, doi:10.1371/journal.pcbi.1002018 (2011).
