# Supplementary material for: Serine synthesis through PHGDH coordinates nucleotide levels by maintaining central carbon metabolism
Source: Nat Commun. 2018 Dec 21;9:5442. doi: 10.1038/s41467-018-07868-6 (PMC6303315; doi:10.1038/s41467-018-07868-6)
Supplement: Supplementary file 3 — Reporting Summary [file 41467_2018_7868_MOESM3_ESM.pdf]

nature research

Corresponding author(s): Jason W. Locasale

Reporting Summary

Nature Research wishes to improve the reproducibility of the work that we publish. This form provides structure for consistency and transparency in reporting. For further information on Nature Research policies, see Authors & Referees and the Editorial Policy Checklist.

Statistical parameters

When statistical analyses are reported, confirm that the following items are present in the relevant location (e.g. figure legend, table legend, main text, or Methods section).

n/a	Confirmed	
☐	☒	The exact sample size (*n*) for each experimental group/condition, given as a discrete number and unit of measurement
☐	☒	An indication of whether measurements were taken from distinct samples or whether the same sample was measured repeatedly
☐	☒	The statistical test(s) used AND whether they are one- or two-sided<br>*Only common tests should be described solely by name; describe more complex techniques in the Methods section.*
☒	☐	A description of all covariates tested
☐	☒	A description of any assumptions or corrections, such as tests of normality and adjustment for multiple comparisons
☐	☒	A full description of the statistics including central tendency (e.g. means) or other basic estimates (e.g. regression coefficient) AND variation (e.g. standard deviation) or associated estimates of uncertainty (e.g. confidence intervals)
☒	☐	For null hypothesis testing, the test statistic (e.g. *F*, *t*, *r*) with confidence intervals, effect sizes, degrees of freedom and *P* value noted<br>*Give P values as exact values whenever suitable.*
☒	☐	For Bayesian analysis, information on the choice of priors and Markov chain Monte Carlo settings
☒	☐	For hierarchical and complex designs, identification of the appropriate level for tests and full reporting of outcomes
☒	☐	Estimates of effect sizes (e.g. Cohen's *d*, Pearson's *r*), indicating how they were calculated
☐	☒	Clearly defined error bars<br>*State explicitly what error bars represent (e.g. SD, SE, CI)*

Our web collection on statistics for biologists may be useful.

Software and code

Policy information about availability of computer code

Data collection	Source code for metabolic flux analysis is available on GITHUB [https://github.com/LocasaleLab/Reid-et-al-2018]
Data analysis	Mass spectrometry peak integration was performed using Thermo SIEVE 2.0 (Thermo). Metabolic flux analysis coding was performed using Python package NumPy. All other data were analyzed using Microsoft Excel and GraphPad Prism.

For manuscripts utilizing custom algorithms or software that are central to the research but not yet described in published literature, software must be made available to editors/reviewers upon request. We strongly encourage code deposition in a community repository (e.g. GitHub). See the Nature Research guidelines for submitting code & software for further information.

Data

Policy information about availability of data

All manuscripts must include a data availability statement. This statement should provide the following information, where applicable:

- Accession codes, unique identifiers, or web links for publicly available datasets
- A list of figures that have associated raw data
- A description of any restrictions on data availability

Raw data for all metabolomics experiments and for metabolic flux analysis is available on GITHUB [https://github.com/LocasaleLab/Reid-et-al-2018].

Field-specific reporting

Please select the best fit for your research. If you are not sure, read the appropriate sections before making your selection.

☒ Life sciences ☐ Behavioural & social sciences ☐ Ecological, evolutionary & environmental sciences

For a reference copy of the document with all sections, see nature.com/authors/policies/ReportingSummary-flat.pdf

Life sciences study design

All studies must disclose on these points even when the disclosure is negative.

Sample size	Three biological replicates were used in all experiments.
Data exclusions	No data were excluded from analyses.
Replication	Experiments were conducted 2-3 times with 3 biological replicates to ensure reproducibility. Experiments were also conducted in at least two unique cell lines.
Randomization	Cells were seeded equally into multiple wells/plates. No preference was given as to which well/plate received which treatment.
Blinding	Investigators were not blinded to group allocation.

Reporting for specific materials, systems and methods

Materials & experimental systems

n/a	Involved in the study	
☐	☒	Unique biological materials
☐	☒	Antibodies
☐	☒	Eukaryotic cell lines
☒	☐	Palaeontology
☒	☐	Animals and other organisms
☒	☐	Human research participants

Methods

n/a	Involved in the study	
☒	☐	ChIP-seq
☒	☐	Flow cytometry
☒	☐	MRI-based neuroimaging

Unique biological materials

Policy information about availability of materials

Obtaining unique materials	The PHGDH inhibitor WQ-2101 was synthesized in Luhua Lai's lab, and will be available upon request.

Antibodies

Antibodies used	Anti-beta-Actin from Cell Signaling (3700S) and anti-PHGDH from Sigma (WH0026227M1)
Validation	According to the manufacturer's website, the anti-beta-Actin antibody is a monoclonal antibody valid for western blotting, immunohistochemistry, immunofluorescence, and flow cytometry. It has been cited in 384 studies. According to the manufacturer's website, the anti-PHGDH antibody is a monoclonal antibody valid for western blot, immunohistochemistry, immunoprecipitation, ELISA, and immunofluorescence.

Eukaryotic cell lines

Policy information about cell lines

Cell line source(s)	HCT116, BT-20, MDA-MB-468, MCF-7, and SCOV3 were purchased from ATCC. MDA-MB-231 were a gift from Dr. Donald McDonnell (Duke University).
Authentication	HCT116, MDA-MB-468, MCF-7, and SCOV3 were authenticated by ATCC. BT-20 and MDA-MB-231 were authenticated by the provider (Anderson et al., Sci Transl Med 2016, PMID: 27974663).

Mycoplasma contamination	No mycoplasma contamination was detected.
Commonly misidentified lines (See ICLAC register)	MCF-7, BT-20
