# Supplementary material for: Serine synthesis through PHGDH coordinates nucleotide levels by maintaining central carbon metabolism
Source: Nat Commun. 2018 Dec 21;9:5442. doi: 10.1038/s41467-018-07868-6 (PMC6303315; doi:10.1038/s41467-018-07868-6)
Supplement: Supplementary file 2 — Peer Review File [file 41467_2018_7868_MOESM2_ESM.docx]

**Editorial Note:** this manuscript has been previously reviewed at another journal that is not operating a transparent peer review scheme. This document only contains reviewer comments and rebuttal letters for versions considered at Nature Communications.

Reviewers' comments:

Reviewer #2 (Remarks to the Author):

In this revised manuscript, the authors have done significant amount of experiments to address previous concerns. The manuscript has been well improved. However, a few questions still exist:

1. The major concern is the specificity of PHGDH inhibitor. Although reviewer figure 1 contains solid data to indicate that WQ-2101 is a potent and specific inhibitor of PHGDH, a discrepancy exists when comparing figure 2 and sup figure 2 to sup figure 4. For instance, WQ-2101 treatment increased citrate/isocitrate, while siPHGDH treatment reduced it; WQ-2101 treatment led to

AMP/ADP/ATP accumulation, while siPHGDH treatment did not… In spite of these differences, siPHGDH treatment did reduced α-KG, R5P and AICAR levels. This is consistent to the finding that ribose+α-KG rescued cells upon PHGDHi treatment. It would be convincing if the authors can test whether ribose+α-KG rescues cells upon siPHGDH treatment.

2. On page 7, in first paragraph it says: ‘Strikingly, we observed flux through the serine synthesis pathway was largely unchanged in response to PHGDH inhibition (Fig 5b-d). In contrast, there was a dramatic reduction in flux through the pentose phosphate pathway (Fig 5e) and the TCA cycle (Fig 5f-k). The same result was found in BT-20 cells (Supplemental Fig 8).’ The authors need to correct this since in sup figure 8 there was also a dramatic reduction in flux through the serine synthesis pathway (possibly because BT-20 cells have high PHGDH expression than HCT116 cells). The results were not exactly ‘same’.
3. The authors still need to test whether ribose+formate rescues cells if they want to claim that ‘PHGDH inhibition alters de novo synthesis of both purine and pyrimidine nucleotides through a mechanism that may occur independent of serine-dependent one carbon metabolism.’ It is possible that disrupting the mass balance will also affect the conversion of serine to one-carbon unit even when serine is not limiting.

Reviewer #3 (Remarks to the Author):

In their manuscript “Serine synthesis coordinates nucleotide levels by maintaining central carbon metabolism” Reid et al. suggest that PHGDH inhibition inhibits nucleotide synthesis by disrupting central carbon metabolism independent of serine utilization. To support their hypothesis, the authors mostly rely on chemical PHGDH inhibition. While this system allows for the interrogation of immediate metabolic changes following PHGDH inhibition, off target effects are a concern. The

authors attempt to show specificity and have included additional data to support these claims, but these concerns are not entirely alleviated. Specific concerns are outlined below:

1. The authors provide new data to compare the metabolite profiles following WQ-2101 treatment and PHGDH siRNA, but the metabolite patterns look very different. While changes in the same pathways are supportive, it would be expected that if these approaches are hitting the same target the bottlenecks would be the same and individual metabolites would change in the same direction. Thus, without additional supporting data I find these data alone insufficient to support the specificity of WQ-2101.
2. Additional experiments to support the on-target effects of WQ-2101 could include:
   a. The use of additional PHGDH inhibitors (e.g. CBR-5884, NCT-503).
   b. The use of an isogenic system to support the specificity of WQ-2101 in this experimental system, and for these pathways. Reconstitution of PHGDH KO cells with WT and R134A mutant enzyme to show that PHGDH that is defective in drug binding, but retains enzyme activity, rescues the metabolite alterations.
3. How much does proliferation influence these metabolic pathways? The authors suggest that PHGDH inhibition influences the activity of the PPP and TCA cycle by affecting flux balance, but might these pathways have decreased flux since the inhibitor causes the cells to stop proliferating altogether (Figure 6B)?

General comments:

We are grateful for the guidance from the editors pertaining to this revision. We are happy to hear that the reviewers believe our manuscript has improved during the revision and that there is interest in the publication of our study in Nature Communications at the editorial level. We also agree completely that the remaining reviewer experimental requests should be addressed. We are thankful for the reviewers’ helpful comments and suggested experiments, the results of which have enhanced the conclusions and scope of our study.

In the revised version of the manuscript, we have further strengthened the specificity of WQ-2101 by performing and including experiments using another published PHGDH inhibitor, NCT-503 (PMID:27110680), and more results using the genetic approach. Specific responses are listed below.

Reviewers' comments:

Reviewer #2 (Remarks to the Author):

In this revised manuscript, the authors have done significant amount of experiments to address previous concerns. The manuscript has been well improved. However, a few questions still exist:

We are thankful for the reviewer’s positive comments that the manuscript has been improved. We are also grateful for the additional comments, which have further enhanced the conclusions.

1. The major concern is the specificity of PHGDH inhibitor. Although reviewer figure 1 contains solid data to indicate that WQ-2101 is a potent and specific inhibitor of PHGDH, a discrepancy exists when comparing figure 2 and sup figure 2 to sup figure 4. For instance, WQ-2101 treatment increased citrate/isocitrate, while siPHGDH treatment reduced it; WQ-2101 treatment led to AMP/ADP/ATP accumulation, while siPHGDH treatment did not… In spite of these differences, siPHGDH treatment did reduced α-KG, R5P and AICAR levels. This is consistent to the finding that ribose+α-KG rescued cells upon PHGDHi treatment. It would be convincing if the authors can test whether ribose+α-KG rescues cells upon siPHGDH treatment.

We appreciate the reviewer’s comments that the siPHGDH and WQ-2101 effects on aKG, R5P, and AICAR levels are consistent. As suggested by the reviewer, the revised manuscript now includes data demonstrating ribose+αKG can rescue cells with PHGDH knocked down (now Supplemental Fig 12a).

To further address the reviewer’s concerns about the compound’s specificity, we have also added new data using another published PHGHD inhibitor, NCT-503 (PMID:27110680), including:

1) steady state 13C-glucose tracing of nucleotide (new Supplemental Fig 5c, 5f)

2) metabolic flux analysis (new Supplemental Fig 7c)

3) kinetic flux profiling experiments (new Supplemental Fig 9)

4) ribose + αKG rescue of cell proliferation (new Supplemental Fig 12b).
2. On page 7, in first paragraph it says: ‘Strikingly, we observed flux through the serine synthesis pathway was largely unchanged in response to PHGDH inhibition (Fig 5b-d). In contrast, there was a dramatic reduction in flux through the pentose phosphate pathway (Fig 5e) and the TCA cycle (Fig 5f-k). The same result was found in BT-20 cells (Supplemental Fig 8).’ The authors need to correct this since in sup figure 8 there was also a dramatic reduction in flux through the serine synthesis pathway (possibly because BT-20 cells have high PHGDH expression than HCT116 cells). The results were not exactly ‘same’.

We agree with the reviewer that the first paragraph on page 7 should be revised, and have done so accordingly:

The sentence (Page 7, lines 7-9) now reads: “Similar reductions in flux through the pentose phosphate pathway and the TCA cycle were found in BT-20 cells treated with WQ-2101 (Supplemental Fig 8) and in cells treated a different PHGDH inhibitor compound, NCT-503 (Supplemental Fig 9)”

3. The authors still need to test whether ribose+formate rescues cells if they want to claim that ‘PHGDH inhibition alters de novo synthesis of both purine and pyrimidine nucleotides through a mechanism that may occur independent of serine-dependent one carbon metabolism.’ It is possible that disrupting the mass balance will also affect the conversion of serine to one-carbon unit even when serine is not limiting.

We agree it is important to test whether disrupting the mass balance upon serine synthesis inhibition affects the conversion of serine to one-carbon unit. We have performed the ribose+formate rescue on cells as suggested by the reviewer, and the new data is now included in the revised manuscript (Supplemental Fig 12c).

Reviewer #3 (Remarks to the Author):

In their manuscript “Serine synthesis coordinates nucleotide levels by maintaining central carbon metabolism” Reid et al. suggest that PHGDH inhibition inhibits nucleotide synthesis by disrupting central carbon metabolism independent of serine utilization. To support their hypothesis, the authors mostly rely on chemical PHGDH inhibition. While this system allows for the interrogation of immediate metabolic changes following PHGDH inhibition, off target effects are a concern. The authors attempt to show specificity and have included additional data to support these claims, but these concerns are not entirely alleviated. Specific concerns are outlined below:

1. The authors provide new data to compare the metabolite profiles following WQ-2101 treatment and PHGDH siRNA, but the metabolite patterns look very different. While changes in the same pathways are supportive, it would be expected that if these approaches are hitting the same target the bottlenecks would be the same and individual metabolites would change in the same direction. Thus, without additional supporting data I find these data alone insufficient to support the specificity of WQ-2101.

We appreciate the reviewer’s comments that changes in the same pathways supports our conclusions that disrupting the serine synthesis pathway affects central carbon and nucleotide metabolism, and have conducted additional experiments to support the specificity of WQ-2101 as suggested by the reviewer below.

2. Additional experiments to support the on-target effects of WQ-2101 could include:
   a. The use of additional PHGDH inhibitors (e.g. CBR-5884, NCT-503).

We are thankful for this suggestion by the reviewer and have conducted new experiments using another published PHGDH inhibitor, NCT-503(PMID:27110680), which provide further evidence that results obtained using WQ-2101 were not due to off-target effects. In the revised manuscript we now included data consistent with the findings in experiments using WQ-2101 including:

1) steady state 13C-glucose tracing of nucleotide (new Supplemental Fig 5c, 5f)

2) metabolic flux analysis (new Supplemental Fig 7c)

3) kinetic flux profiling experiments (new Supplemental Fig 9)

4) ribose + aKG rescue of cell proliferation (new Supplemental Fig 12b).
   b. The use of an isogenic system to support the specificity of WQ-2101 in this experimental system, and for these pathways. Reconstitution of PHGDH KO cells with WT and R134A mutant enzyme to show that PHGDH that is defective in drug binding, but retains enzyme activity, rescues the metabolite alterations.

We agree with the reviewer that an isogenic system with a WT and mutant PHGDH that is defective in drug binding but retains enzyme activity would be useful. WQ-2101 binds PHGDH at an allosteric site near the NAD+ binding domain, which is an essential co-factor, thus we suspect it would be difficult to find a mutant that has reduced WQ-2101 binding while retaining enzyme activity. For example, while the R134A reduces binding of WQ-2101, it also reduces the overall enzyme activity (Reviewer Fig 1).

3. How much does proliferation influence these metabolic pathways? The authors suggest that PHGDH inhibition influences the activity of the PPP and TCA cycle by affecting flux balance, but might these pathways have decreased flux since the inhibitor causes the cells to stop proliferating altogether (Figure 6B)?

We believe the changes in TCA cycle and PPP flux into nucleotides are what causes the defects in cell proliferation. In support of this, cells with PHGDH inhibition have similar cell numbers at 6 hours compared to control, yet already display alterations to central carbon and nucleotide metabolism (Reviewer Fig 2).

REVIEWERS' COMMENTS:

Reviewer #2 (Remarks to the Author):

The authors have used another PHGDH inhibitor NCT503 and siRNA against PHGDH as independent methods to prove the reproducibility and robustness of their findings. The previous concerns are fully addressed.

Reviewer #3 (Remarks to the Author):

The authors cannot conclude based on cell counts at 6 hours that proliferation is not affected at this time point. Because of the long doubling time of mammalian cells compared to bacteria, for example, cell counts would not be expected to change by 6 hours even if cells were completely arrested at time 0. BrdU labeling to analyze number of cells in S phase would be more appropriate. The authors either need to suitably show that 1.) metabolite changes precede proliferation changes, or 2.) inhibition of proliferation does not induce the same metabolic changes, or 3.) add this point to the discussion.

General comments:

We are grateful for the guidance from the editors pertaining to this revision. We are happy to hear that Reviewer 2’s concerns have been fully addressed. We also agree with Reviewer 3 that it is important to comment in the discussion on how the proliferation status of the cells may impact metabolic phenotypes. We are thankful for the reviewers’ helpful comments, the results of which have enhanced the conclusions and scope of our study.

REVIEWERS' COMMENTS:

Reviewer #2 (Remarks to the Author):

The authors have used another PHGDH inhibitor NCT503 and siRNA against PHGDH as independent methods to prove the reproducibility and robustness of their findings. The previous concerns are fully addressed.

We are happy to hear that the reviewer was satisfied with our response.

Reviewer #3 (Remarks to the Author):

The authors cannot conclude based on cell counts at 6 hours that proliferation is not affected at this time point. Because of the long doubling time of mammalian cells compared to bacteria, for example, cell counts would not be expected to change by 6 hours even if cells were completely arrested at time 0. BrdU labeling to analyze number of cells in S phase would be more appropriate. The authors either need to suitably show that 1.) metabolite changes precede proliferation changes, or 2.) inhibition of proliferation does not induce the same metabolic changes, or 3.) add this point to the discussion.

The reviewer raises an important consideration. Several recent studies have found restoring nucleotide metabolism rescues proliferative defects such as those resulting from autophagy deficiency (Guo et al. Genes&Dev 2016, PMID: 27516533), PKM1 expression (Lunt et al. Mol Cell 2015, PMID: 25482511), mutant p53 knockdown (Kollareddy et al. Nat Comm 2015, PMID: 26067754), and PI3K inhibition (Juvekar et al. PNAS 2016, PMID: 27402769). Among these studies, cell cycle was only explored upon PI3K inhibition and, interestingly, they found reduced glycolytic flux preceded proliferation arrest as demonstrated by cells still entering S phase upon PI3K inhibition while glycolytic flux was already reduced. Moreover, Bester et al. (Cell 2011, PMID: 21529715) describe a mechanism whereby oncogenic growth signaling triggers cell division without coordinate nucleotide synthesis, which promotes genome instability but can be rescued by nucleoside supplementation.

To address this comment, we have added the following to the discussion (page 9, line 5-8):

“Our findings are thought to be a direct mechanism of PHGDH inhibition rather than a general effect of reduced cell proliferation, consistent with previous reports showing metabolic flux and metabolite availability may not coordinate with proliferation status^22,23^.”
